# 1200 high-quality metagenome-assembled genomes from the rumen of African cattle and their relevance in the context of sub-optimal feeding

**DOI:** 10.1186/s13059-020-02144-7

**Published:** 2020-09-03

**Authors:** Toby Wilkinson, Daniel Korir, Moses Ogugo, Robert D. Stewart, Mick Watson, Edith Paxton, John Goopy, Christelle Robert

**Affiliations:** 1 The Roslin Institute and R(D)SVS, University of Edinburgh, Easter Bush, Roslin, EH25 9RG UK; 2 International Livestock Research Institute (ILRI), P.O. Box 30709, Nairobi, 00100 Kenya

## Abstract

**Background:**

The Boran (*Bos indicus*), indigenous Zebu cattle breed from sub-Saharan Africa, is remarkably well adapted to harsh tropical environments. Due to financial constraints and low-quality forage, African livestock are rarely fed at 100% maintenance energy requirements (MER) and the effect of sub-optimal restricted feeding on the rumen microbiome of African Zebu cattle remains largely unexplored. We collected 24 rumen fluid samples from six Boran cattle fed at sub-optimal and optimal MER levels and characterised their rumen microbial composition by performing shotgun metagenomics and de novo assembly of metagenome-assembled genomes (MAGs). These MAGs were used as reference database to investigate the effect of diet restriction on the composition and functional potential of the rumen microbiome of African cattle.

**Results:**

We report 1200 newly discovered MAGs from the rumen of Boran cattle. A total of 850 were dereplicated, and their uniqueness confirmed with pairwise comparisons (based on Mash distances) between African MAGs and other publicly available genomes from the rumen. A genome-centric investigation into sub-optimal diets highlighted a statistically significant effect on rumen microbial abundance profiles and a previously unobserved relationship between whole microbiome shifts in functional potential and taxon-level associations in metabolic pathways.

**Conclusions:**

This study is the first to identify 1200 high-quality African rumen-specific MAGs and provides further insight into the rumen function in harsh environments with food scarcity. The genomic information from the rumen microbiome of an indigenous African cattle breed sheds light on the microbiome contribution to rumen functionality and constitutes a vital resource in addressing food security in developing countries.

## Background

Ruminant livestock represent an important part of human nutrition as a major source of our meat and milk [1]. Increase in population and demand for such products means that by 2050, production of meat and milk must increase by 76% and 63% globally, if demands are to be met [2]. This presents novel challenges for the African livestock sector where increase in demand exceeds population growth, showing a greater correlation to an increase in disposable income. Herrero and colleagues [3] highlighted that investigation into the current and long-term genetic potential of indigenous livestock breeds will help address some of these challenges. The rumen is a complex anaerobic microbial ecosystem which plays an essential role in feed digestion through microbial fermentation. The functionality of the rumen microbial population allows the conversion of plant material of relatively low nutritional value into readily absorbed vital compounds for the animal. Furthermore, the efficiency of the different fermentation processes employed by the rumen microbial community dictates the quality and quantity of production within each animal [4–6]. In commercial settings, ruminant production as a product of rumen microbiology is a widely studied area of agricultural science. Surveys of smallholder famers in sub-Saharan Africa have shown that nearly 60% of farmers in these production systems consider lack of forage as one of the major constraints they face [7]. Despite this, investigation in sub-optimal farming environments is still underrepresented. Understanding the contribution of all members of the microbiome to rumen functionality is paramount to the development of feeding and farming strategies to maximise animal production to support small farming communities and preserve global food security. Given this necessity, organisms from the rumen microbiome, regardless of host species and location, are generally underrepresented in genome and marker gene databases. To address this shortfall, the 16S databases have been added to by projects such as the Global Rumen Census [8], while the Hungate1000 has cultured and sequenced more than 400 rumen microbial genomes [9]. Although the Hungate collection represents a global effort, only six of the isolates originated from the African continent all from ovine origin. Furthermore, despite the Hungate1000 project improving the availability of genomic information for the 88 described rumen genera from 12.5 to 83% [9] as well as adding 30 genomes from the list of 70 ‘most wanted’ rumen genomes, this only serves to highlight the limitations of current culturing strategies. Furthermore, while efforts have been made to address the lack of genomic information about rumen organisms [9], and then use newly sequenced genomes to infer rumen function [10], metagenome-assembled genomes represent a set of environment-specific genomes that has not been biassed by culture or isolation techniques [9].

Recent work by Stewart et al. [11, 12] has shown the value of culture-free methods in the isolation of genomic information for rumen organisms through the release of 913 and then 4941 metagenome-assembled genomes (MAGs), leading to a fivefold increase in the taxonomic classification of rumen taxa from various other rumen studies. The authors also provided an unparalleled resource of functional information in the form of thousands of novel carbohydrate active enzymes (CAZymes). As a tool for the further study of the rumen environment, MAG data has already allowed further insight into the complex suite of pathways involved in carbon degradation, even in previously unknown taxa and lineages [11, 13]. However, as previously highlighted, the 913 MAGs (referred to as RUGs [11]) originate from the rumen of 43 Scottish cattle, the 4194 MAGs (referred to as RUG 2.0 genomes [12]) from an additional 240 Scottish cattle—all consisting of three cross breeds (Aberdeen Angus, Limousin and Charolais) and one pure breed (Luing), and do not represent the diversity of the rumen ecosystem in cattle in other geographical locations and under distinct feeding regimes [8].

To address the underrepresentation of genomes from the rumen of African cattle living in harsh tropical environments, we aimed at identifying the specificity and uniqueness of the rumen microbiota in indigenous cattle exposed to food scarcity. Here, we identified and characterised novel MAGs extracted from rumen samples of Boran cattle in sub-Saharan Africa that were either fed at the minimum energy requirement (MER) or subjected to feed restriction (40%, 60% and 80% MER). We used a genome-centric approach to estimate MAG abundance and further investigate the MAG’s functional potential [14]. The new MAGs extend the genomic resources currently available to the scientific rumen microbiome community. The genomic information from the rumen microbiome of an indigenous African cattle breed presented here further helps in deciphering the microbiome contribution to rumen functionality in African cattle species and provides a vital resource in addressing food security in developing countries.

## Results

### Sequencing and assembly

**Fig. 1:**
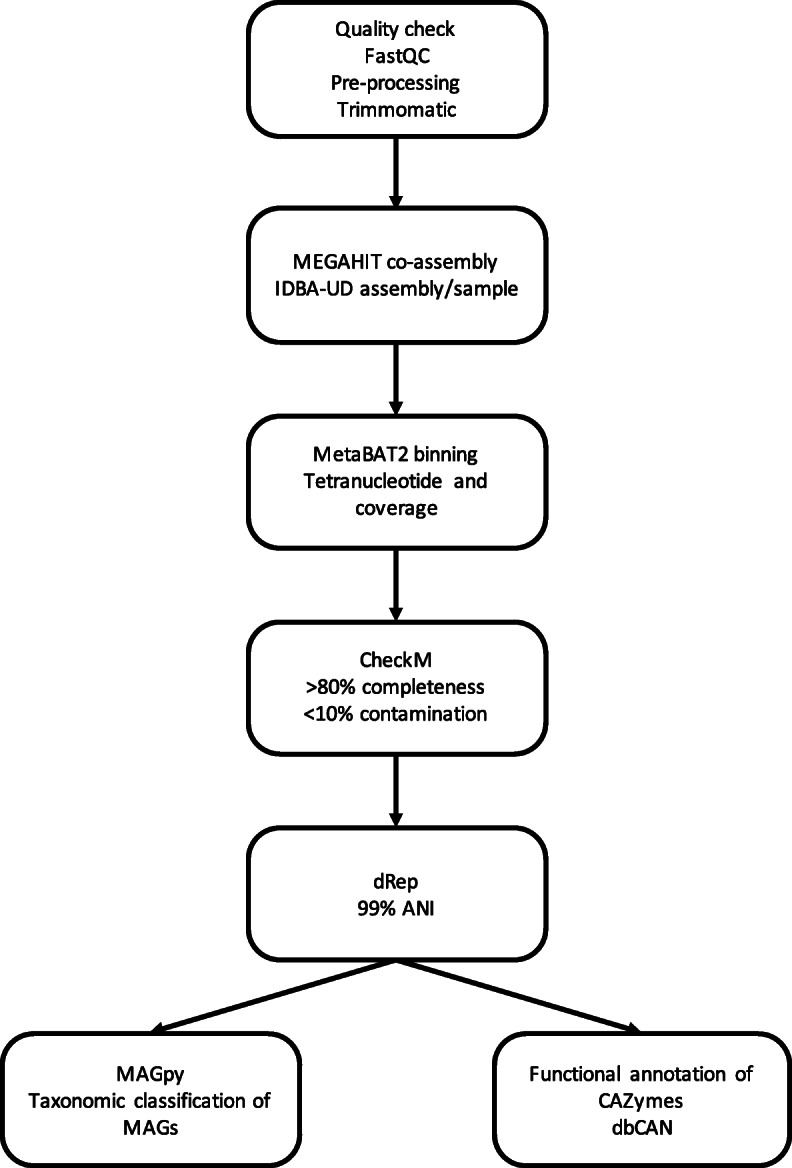
Bioinformatics workflow. Sequence processing and MAG discovery and identification workflow adapted from Stewart et al. [11] (additional details in text)

**Fig. 2:**
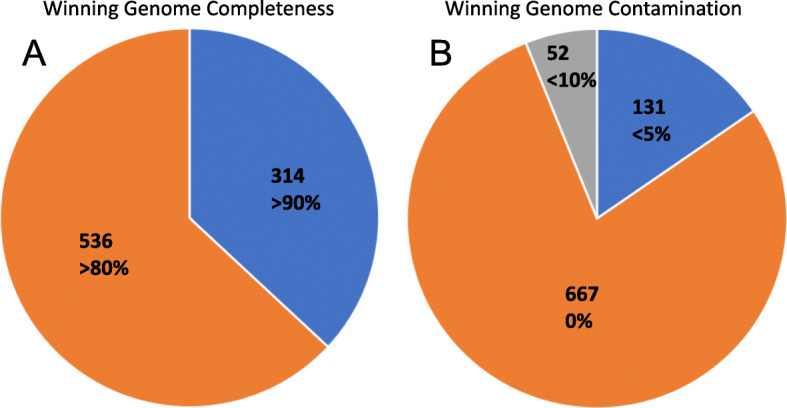
CheckM-generated quality metrics for all 850 dereplicated African MAGs. **a** Distribution of completeness. Orange: substantial completeness (> 80%). Blue: near completeness (> 90%). **b** Distribution of contamination level. Grey: medium (< 10%) contamination. Blue: low (< 5%) contamination. Orange: no contamination

Sequencing on an Illumina HiSeq 4000 generated 749 Gb of data from 24 samples from the rumen of African Zebu cattle. Following read trimming, using Trimmomatic [15], the samples contain an average of 106 million 150-bp paired-end reads (ranging from 67 to 255 M). Single-sample assembly and co-assembly were performed using IDBA-UD [16] and MEGAHIT [17] as described in the workflow (see Fig. 1 and the ‘Methods’ section). Binning of the resulting contigs from single-sample assembly and co-assembly produced 7040 bins and 3623 bins, respectively, for a total of 10,663 draft-quality metagenome-assembled genomes (MAGs). Filtering those MAGs based on completeness and contamination using CheckM [18] resulted in 1200 high-quality MAGs. Following dereplication using dRep [19], 850 ‘winning’ MAGs remained (Fig. 2), i.e. 350 MAGs showed greater than 99% similarity to another MAG and had a lower genome quality score as calculated with dRep. Additional clustering based on at least 99% similarity, as described in the ‘Methods’ section, highlighted that 616 of the clusters represented singletons (51.3%), while a further 165 clusters contained duplicate bins accounting for a further 27.5% of all high-quality bins (Table 1). Interestingly, none of the MAGs was 100% identical to each other, and therefore, all 1200 MAGs contain unique genetic information. The 850 winning genomes represent 697 from the single-sample assemblies and 153 from the co-assembly.

**Table 1:** Distribution of 1200 high-quality MAGs in secondary clusters from the dRep workflow

#MAGs (cluster size)	No. of clusters	No. of genomes (%)
7	3	21 (1.75)
6	3	18 (1.5)
5	7	35 (2.92)
4	12	48 (4)
3	44	132 (11)
2	165	330 (27.5)
1	616	616 (51.33)
Total	850	1200

The three largest clusters consisted of 7 genomes, indicating that in this case 21 high-quality genomes were dereplicated into 3 ‘winning’ genomes. Singleton clusters (unique genomes) accounted for 616 genomes, and doubletons more than a quarter of all 1200 MAGs

### Taxonomic classification

**Fig. 3:**
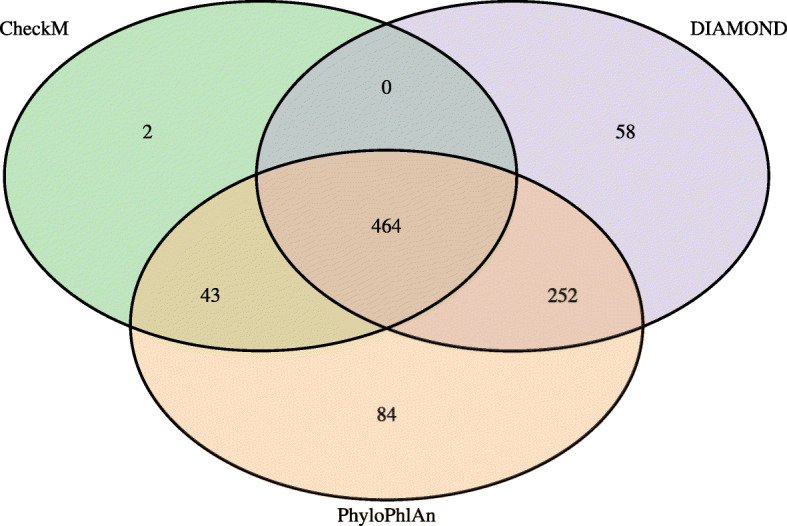
Venn diagram showing numbers of taxonomic assignments in agreement between methods for 850 MAGs. Classification of the taxonomic assignments of the 850 winning MAGs by the three methods incorporated in MAGpy (CheckM, DIAMOND, PhyloPlAn). Intersections represent numbers of taxonomic classification in agreement between methods

For basic prokaryotic classification of MAGs, the CheckM workflow within MAGpy successfully [20] classified all 850 MAGs whereas DIAMOND and PhyloPhlAn classified 809 and 843, respectively. Moving down the taxonomic ranks, CheckM classifies the lowest at each subsequent rank. PhyloPhlAn classifies the most MAGs down to order level but only 159 and 51 at family and genus levels, respectively. The DIAMOND method classifies 594 and 592 MAGs at family and genus levels (Fig. 3 and Table S1). As the DIAMOND classification resolved the taxonomic classification of more bins at lower taxonomic ranks, the results of this classification method were used for further comparisons.

Of the 1200 high-quality MAGs, 385 (32.08%) were classified as members of the *Bacteroidetes* phylum, 514 (42.83%) as *Firmicutes* and 85 (7.08%) as *Proteobacteria* (Table 2)*.* The proportions of genomes classified as members of each of the identified rumen phyla were significantly, highly correlated to those from RUG (Pearson’s *r* = 0.99, *P* < 0.001) and RUG 2.0 (Pearson’s *r* = 0.98, *P* < 0.001) genomes. Spearman’s rank correlation of the same phylum-level classifications indicated that the relative proportions of the major phyla were similar between the African MAG and RUG (*r* = 0.79) and RUG 2.0 (*r* = 0.63) genomes, but that some of the phyla with lower abundance may represent more variable proportions of the microbiome, while the major difference is the relative proportion of *Lentisphaerae* genomes identified (Table 2).

**Table 2:** Phylum-level taxonomic classification of MAGs and RUGs

Classified phyla	High-quality MAGs (1200)		Winning MAGs (850)		Scottish RUGs (850)		RUG 2.0 (4941)	
	No. of genomes	%	No. of genomes	%	No. of genomes	%	No. of genomes	%
Bacteroidetes	385	32.08	253	29.76	288	33.88	1707	34.55
Firmicutes	514	42.83	377	44.35	391	46.00	2407	48.71
Proteobacteria	85	7.08	65	7.65	30	3.53	133	2.69
Fibrobacteres	3	0.25	2	0.24	6	0.71	42	0.85
Actinobacteria	3	0.25	2	0.24	30	3.53	164	3.32
Lentisphaerae	76	6.33	50	5.88	8	0.94	20	0.40
Spirochaetes	7	0.58	6	0.71	10	1.18	59	1.19
Tenericutes	22	1.83	16	1.88	4	0.47	118	2.39
Planctomycetes	9	0.75	5	0.59	4	0.47	12	0.24
Fusobacteria	5	0.42	4	0.47	1	0.12	0	0.00
Verrucomicrobia	1	0.08	1	0.12	0	0.00	47	0.95
Elusimicrobia	16	1.33	13	1.53	8	0.94	26	0.53
Synergistetes	1	0.08	1	0.12	0	0.00	0	0.00
Ignavibacteriae	1	0.08	1	0.12	0	0.00	0	0.00
Euryarchaeota	7	0.58	6	0.71	29	3.41	126	2.55
Bacteria	65	5.42	48	5.65	41	4.82	80	1.62

Distribution of genomes classified to prokaryotic genera within the high-quality (1200) and ‘winning’ (850) MAGs, metagenome-assembled original Scottish RUGs and RUG 2.0 genomes

### Uniqueness

**Fig. 4:**
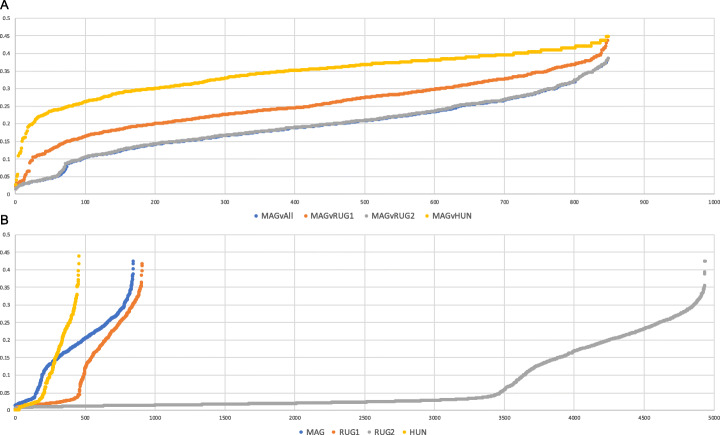
Distribution of minimum Jaccard distance (*y* axis) between sets of genomes. Distances within a single dataset from African MAGs (MAG), Scottish RUGs (RUG) or Hungate (HUN); between two datasets (MAGvRUG, MAGvHUN, RUGvHUN, MAGvAll, RUGvAll, HUNvAll). *X* axis shows number of genomes

**Fig. 5:**
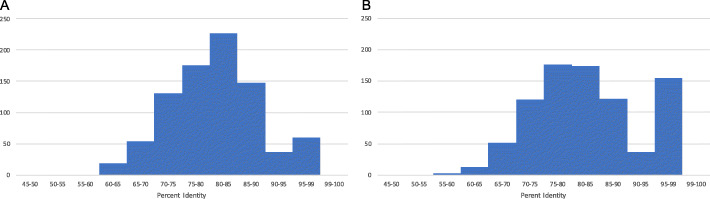
Number of MAGs with maximum percent identities calculated from Jaccard Mash distances. **a** 850 MAGs compared to 913 RUGs, 4941 RUG 2.0 and 460 Hungate genomes. **b** 850 MAGs compared to all other MAGs

Following dereplication with the dRep pipeline, all 850 African MAGs and 913 Scottish RUGs were identified as ‘winning’ genomes and were therefore not more than 99% similar to another genome. Additionally, pairwise MASH [21] comparison of all MAGs, RUGs, Hungate and RUG 2.0 genomes shows that no African MAGs show complete identity to any other publicly available rumen organism genome, and only 97 MAGs show at least 90% similarity to any other genome, 93 compared to RUG 2.0 and 3 compared to genomes from the Hungate collection and only one from the original Scottish RUGs (Figs. 4 and 5a and Table S2), and the majority of the MAGs (753/850) show less than 90% similarity to any of the publicly available genomes (Fig. 5a). Comparing the genomes within the African MAG dataset, 658 MAGs show less than 90% similarity to another MAG, and 192 genomes showing at least 90% similarity to another MAG (Figs. 4and 5b and Table S2).

### Functional annotation

Comparison of the 1,613,102 predicted proteins to the KEGG database resulted in a total of 768,535 hits to 3999 KEGG orthologs (KOs) identified in the African MAGs. These mapped to 7470 pathway modules within the KEGG BRITE database (Table S3 – KO_Counts). Comparison of the KOs identified within the African MAGs to those within the CowPI database [10], which consists of 4585 KOs from genomes in the Hungate collection, highlighted 2675 KOs in common between these datasets. The CowPI data contained 1910 KOs not identified in the African MAGs, 1049 of which are associated with metabolism functions. The African MAGs contained 1324 KOs that have not been identified in the CowPI dataset, 573 of which are associated with metabolism. The MAGs contained 260 KOs, not present in the Hungate genomes, that are poorly characterised within the KEGG database, whereas the Hungate genomes only contain 53 such KOs (Table S3 – KO_Counts).

Comparison of MAG proteins to HMM profiles representing CAZymes, as provided in the dbCAN [22] database, resulted in identification of 69,628 carbohydrate active enzyme sites across 68,850 proteins within the dataset, consisting of 39,566 glycoside hydrolases (GH), 17,473 glycosyl transferases (GT), 1052 polysaccharide lyases (PL), 9060 carbohydrate esterases (CE), 111 with auxiliary activity (AA) and 2366 carbohydrate binding modules (CBM) (Table S4 – CAZy_Counts).

**Fig. 6:**
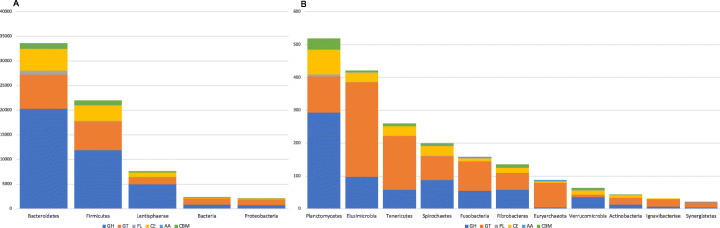
Total counts of enzymes in each CAZy module as assigned by dbCAN. Counts have been aggregated to represent the representation of each module in each of the major (**a**) and minor (**b**) phyla of the rumen

**Fig. 7:**
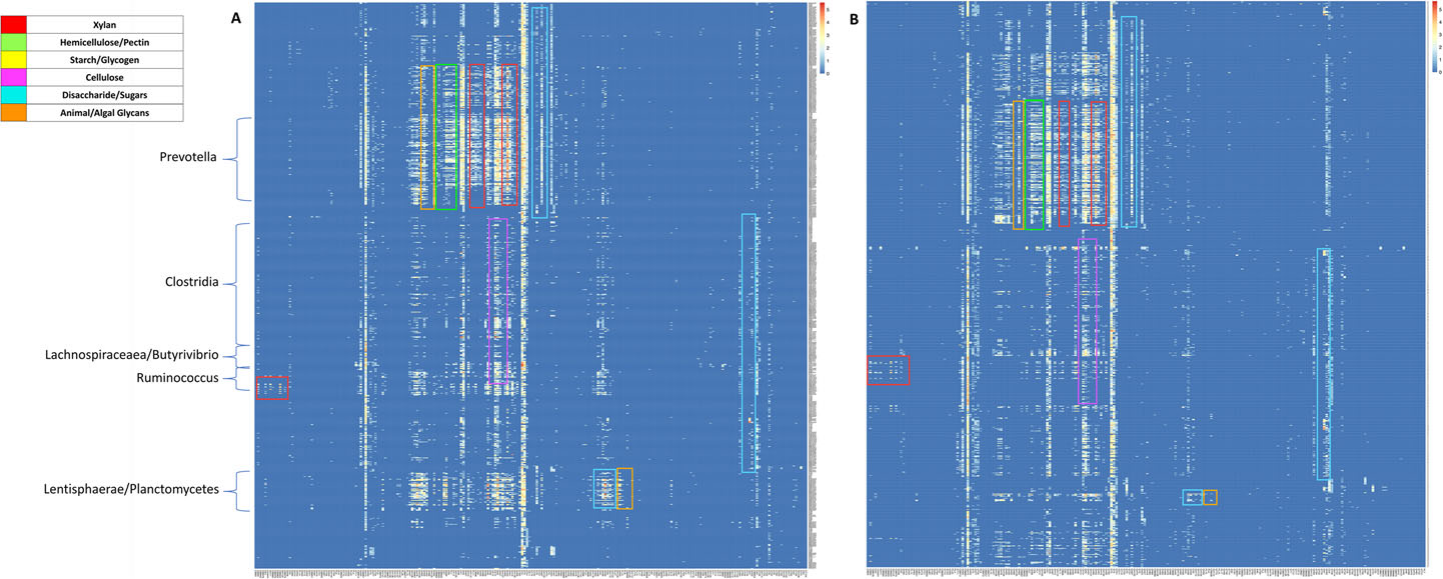
Heatmaps displaying counts of enzymes belonging to each CAZy family, in each MAG. Clusters of CAZyme families involved in the breakdown of selected polysaccharides are highlighted by coloured boxes. Heatmaps generated from CAZymes present in African MAGs (**a**) or Scottish RUGs (**b**)

The proportion of CAZymes identified within African MAGs shows a similar distribution to those identified in the both the original RUGs from Scottish cattle and RUG 2.0 (Table 3). CAZymes also showed similar taxonomic association with MAGs/RUGs identified as being taxa involved with carbohydrate metabolism (Table 4, Figs. 6 and 7).

**Table 3:** Presence and distribution of CAZy modules within the MAGs and RUGs

CAZy module	African MAGs		Scottish RUGs		RUG 2.0	
	Count	%	Count	%	Count	%
GH	39,566	56.82	40,140	55.14	235,001	53.09
GT	17,473	25.09	19,722	27.09	120,494	27.22
PL	1052	1.51	1121	1.54	6834	1.54
CE	9060	13.01	9119	12.53	55,523	12.54
AA	111	0.16	154	0.21	907	0.20
CBM	2366	3.40	2545	3.50	23,928	5.41

Counts of enzymes identified in each CAZyme module within the entire MAG and RUG datasets. *GH* glycoside hydrolase, *GT* glycosyl transferase, *PL* polysaccharide lyases, *CE* carbohydrate esterases, *AA* auxiliary activities, *CBM* carbohydrate binding modules

**Table 4:** Distribution of CAZy modules across the different MAG taxa

Phylum	GH %	GT %	PL %	CE %	AA %	CBM %
Bacteroidetes	60.44	20.61	2.49	13.12	0.00	3.34
Firmicutes	54.41	26.65	0.51	14.19	0.23	4.01
Lentisphaerae	65.04	18.89	1.08	11.98	0.30	2.72
Bacteria	36.05	52.36	0.13	9.55	0.04	1.88
Proteobacteria	37.27	50.76	0.46	8.44	0.51	2.55
Planctomycetes	56.76	21.04	1.16	14.48	0.39	6.18
Elusimicrobia	23.33	68.33	0.24	7.14	0.71	0.24
Tenericutes	22.31	63.08	0.00	11.54	0.00	3.08
Spirochaetes	44.22	36.18	1.01	15.08	2.01	1.51
Fusobacteria	34.81	56.33	1.27	5.06	2.53	0.00
Fibrobacteres	42.96	38.52	0.00	11.85	0.00	6.67
Euryarchaeota	5.68	84.09	0.00	5.68	4.55	0.00
Verrucomicrobia	56.25	10.94	0.00	21.88	1.56	9.38
Actinobacteria	30.23	46.51	0.00	20.93	0.00	2.33
Ignavibacteriae	22.58	74.19	0.00	3.23	0.00	0.00
Synergistetes	18.18	72.73	0.00	0.00	9.09	0.00

Proportions of CAZymes assigned to each CAZy module within MAGs classified as each of the rumen phyla

To assess novelty, all 68,850 identified carbohydrate active proteins from African MAGs were compared to proteins in the nr, env_nr, M5nr and UniProt/TrEMBL databases; the Hess et al. gene predictions; and additionally the RUG and RUG 2.0 predicted proteins. Only 1286 proteins were completely identical to a protein in any other database or dataset. Furthermore, 78.2% (53,838) of proteins with carbohydrate activity showed less than 95% protein identity to a protein in any other database or dataset (Table 5).

**Table 5:** Novelty of CAZyme proteins

Protein set	PID (% protein identity) thresholds					
	100%	99%	95%	90%	85%	80%
nr	9	93	402	960	2020	3653
env_nr	1	1	11	33	210	661
M5nr	1	2	96	532	1764	4073
UniProt/TrEMBL	2	8	83	415	1169	2468
Hess	839	1553	5404	10,947	17,597	24,151
RUG1	116	665	3058	7173	12,489	18,590
RUG2	338	2028	8243	17,342	26,825	35,159
Total unique	1286	4172	15,012	28,561	40,458	49,323

Counts of hits at distinct minimum percentages of protein identity (PID) thresholds (100%, 99%, 95%, 90%, 85% and 80%) of all 68,850 carbohydrate active proteins from African MAGs against nr, env_nr, M5nr and UniProt/TrEMBL databases and the Hess et al. (Hess), original RUG (RUG1) and RUG 2.0 (RUG2) predicted proteins. The total unique number represents the number of proteins from the African MAGs that have a hit in at least one database

Comparison of CAZyme families present in each dataset indicated the RUGs contained 218 CAZymes in total with 8 GHs, 7 CBMs, 2 GTs and one AA and one PL only encoded by the RUGs, whereas the MAGs contained 225 total CAZymes and 13 GHs, 3 CBMs, 7 GTs and 3 PLs not present in the RUGs. The RUG 2.0 genomes encoded 224 of the 225 MAG CAZymes, missing GH100 as in the RUGs. They also encoded all 19 of the CAZymes contained in the RUGs and not the MAGs, and an additional 22 CAZymes not found in the MAGs or RUGs. Clustering reconstructed groups of enzymes associated with taxa known to contribute to specific substrate degradation, for example, cellulose degradation in the Clostridia, xylan in the Prevotellaceae and Ruminococcaceae and hemicellulose also in the Prevotella (Fig. 7). Comparison of the clustered CAZyme profile of African MAGs (Fig. 7a, Fig. S1) to that of the Scottish RUGs (Fig. 7b, Fig. S2) indicates differences in the abundance of some enzyme families within the genomes of rumen organisms from the two systems.

### Effect of diet restriction on rumen microbiome structure and functional potential

Estimation of MAG abundance by mapping reads to MAG contigs, and correcting for length and GC content bias, showed that all 850 MAGs were present in all animals and all diets, with at least one supporting read (Table S5). Average abundance of the major rumen phyla associated with each diet treatment ranged between 53.15–57.80% for the *Bacteroidetes*, 21.54–24.28% for the *Firmicutes* and 2.52–3.41% of *Proteobacteria*. Additionally, the abundance of MAGs classified as members of the phylum *Lentisphaerae* ranged between 12.03 and 12.78% (Table 6). Analysis at MAG level indicated 30 (20 log2 fold change (LFC) > 0, 10 LFC < 0) differentially abundant MAGs when comparing 40% and 80% MER diet treatments, and 30 (16 LFC > 0, 14 LFC < 0) when contrasting 60 to 80% MER diets (Table S6). Classification of the differentially abundant MAGs highlighted that the same 30 MAGs were differentially abundant when contrasting 40% and 80% MER diet and when contrasting the 60 to 80% MER diets. Of the 30 differentially abundant MAGS, 5 were identified as *Clostridiales*, 4 as *Clostridium*, 1 as *Bacteroides*, 7 as *Bacteroidales*, 3 as *Prevotella* and 2 as *Ruminococcus* (Table S6).

**Table 6:** Estimated relative abundance of MAGs classified at phylum level across diet treatments

Phylum	Average relative abundance (%)		
	40% MER	60% MER	80% MER
Bacteroidetes	53.15	57.80	55.31
Firmicutes	24.28	21.54	24.12
Proteobacteria	3.41	2.52	2.93
Fibrobacteres	0.08	0.24	0.10
Actinobacteria	0.02	0.01	0.01
Lentisphaerae	12.03	12.78	12.74
Spirochaetes	0.51	0.34	0.24
Tenericutes	1.02	0.57	1.41
Planctomycetes	1.33	0.47	0.28
Fusobacteria	0.43	0.07	0.33
Verrucomicrobia	0.003	0.010	0.043
Elusimicrobia	2.93	3.23	1.87
Synergistetes	0.0240	0.0016	0.0004
Ignavibacteriae	0.21	0.10	0.25
Euryarchaeota	0.12	0.08	0.10
Bacteria	0.46	0.23	0.26

Estimated relative abundance of MAGs classified by MAGpy (DIAMOND) as members of rumen phyla. Values represent average abundance of each phylum associated with each diet treatment (40%, 60% and 80% MER)

**Fig. 8:**
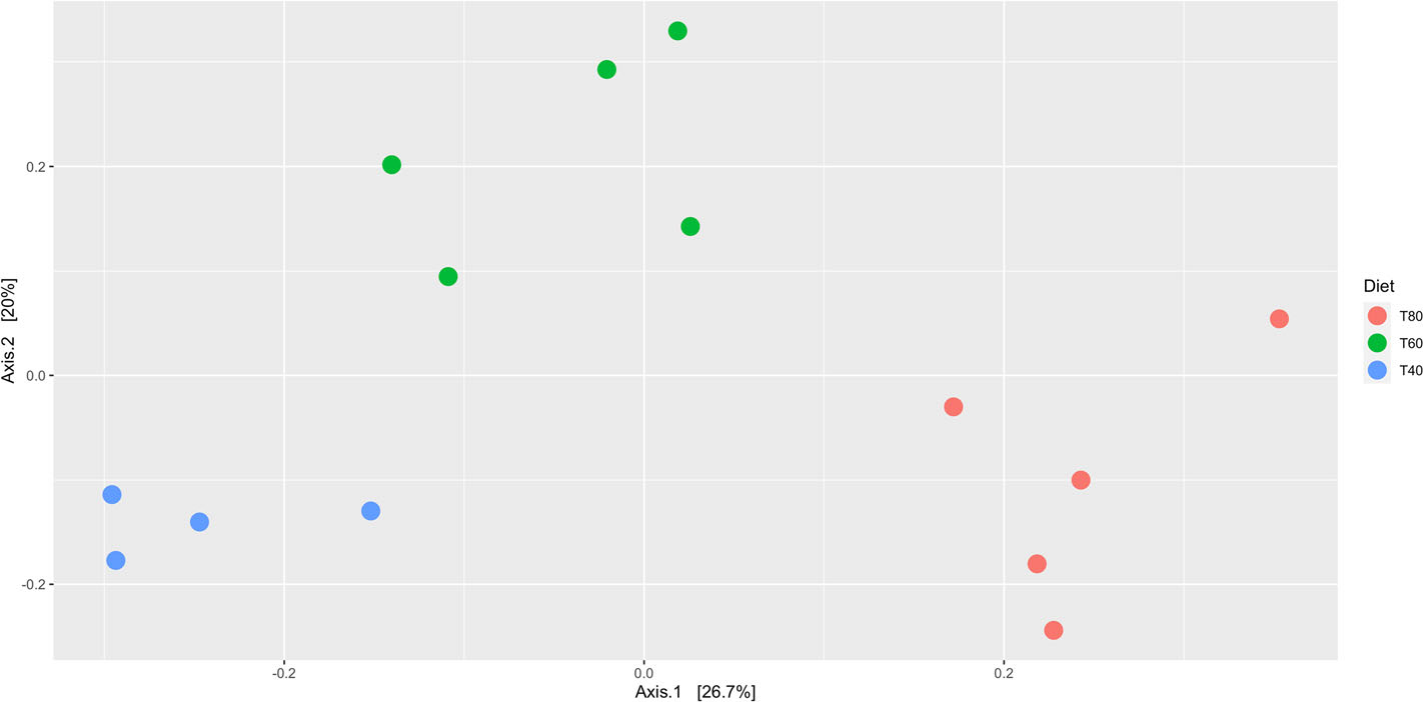
Principal coordinate analysis (PCoA) ordination based on Euclidean distance between samples. Distance between samples was calculated from estimated MAG abundance. PCoA shows distinct clustering based on association with each diet treatment

Principal coordinate analysis (PCoA) of Euclidean distances between samples calculated from Hellinger transformed MAG abundance estimates that were batch corrected for animal and period effects indicates highly distinct clustering of samples associated with each diet. The first principal coordinate (Axis.1 in Fig. 8) accounts for 26.7% of the variance between samples and shows the greatest distinction between clusters of samples representing the 40% and 80% diets. The second principal coordinate (Axis.2 in Fig. 8) accounts for 20% of the variance and distinguishes samples representing the 60% diet from all other samples. After 9999 permutations, Adonis2 (PERMANOVA) analysis on the same Hellinger transformed and batch corrected data indicates that rumen community structure is highly significant (*P* = 0.0001) with regard to the diet, as well as significant (*P* < 0.05) in all pairwise comparison between each diet.

**Fig. 9:**
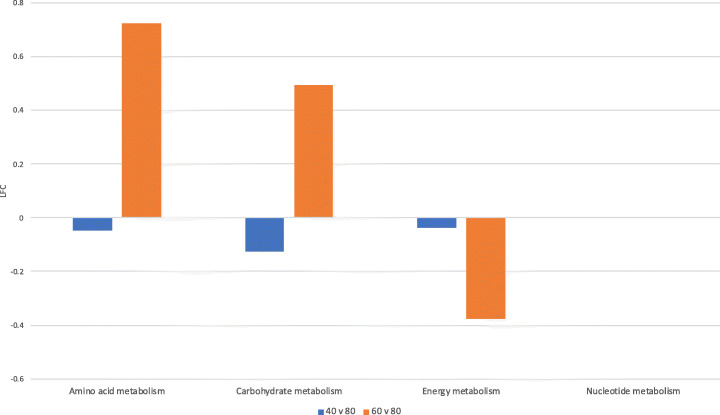
Fold changes of significantly differentially abundant KOs. Contrasting functional potential in the rumen metagenomes associated with 40% vs 80% MER diets in blue, and 60% vs 80% MER diets in orange. Bars represent log2 fold changes (LFC) of significantly differentially abundant KOs associated with amino acid metabolism, carbohydrate metabolism, energy metabolism and nucleotide metabolism

To assess the effect of diet on the functional potential of the rumen microbiome of African cattle, KO counts per MAG (Table S3) were multiplied by the abundance of each MAG per sample (Table S5) to generate a KO per sample matrix to represent the functional potential of the rumen microbiome of each sample (Table S7). Comparison of KOs associated with the 40% MER diet and 60% MER diet against the 80% MER diet revealed significant differential abundances of 147 (91 LFC > 0, 56 LFC < 0, Table S8 – 40vs80) and 147 (78 LFC > 0, 69 LFC < 0, Table S8 – 60vs80) KOs, respectively. Classification of the differentially abundant KOs indicated that the same 147 KOs were differentially abundant between the 40% and 80% diets and between the 60% and 80% diets. Of the 147, 54 (36.7%) are directly involved in metabolism pathways. The fold changes of significantly differentially abundant KOs associated with the amino acid metabolism, carbohydrate metabolism, energy metabolism and nucleotide metabolism pathway modules are highlighted in Fig. 9.

#### Interplay of significantly differential abundant genomes with significant change in functional potential

**Fig. 10:**
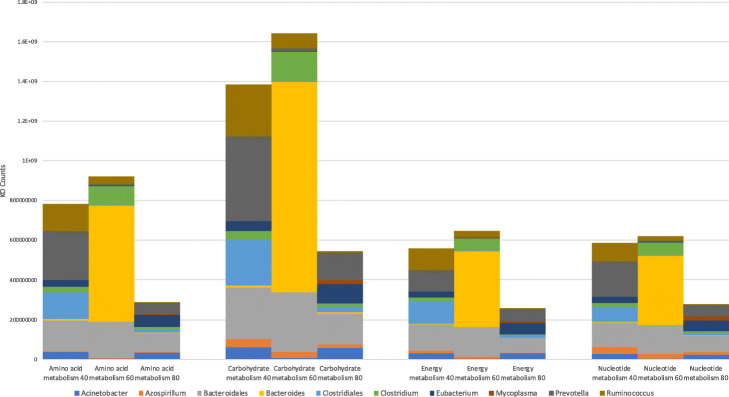
Contribution to metabolic pathways by differentially abundant MAGs. Counts of KOs associated with amino acid metabolism, carbohydrate metabolism, energy metabolism and nucleotide metabolism, encoded by the 10 classified groups of differentially abundant MAGs between 40%, 60% and 80% MER diets

To highlight the power of our genome-centric approach in the analysis of the rumen metagenome, significant changes in the overall functional potential of the microbiome were contrasted with differences accounted for by the significantly differentially abundant genomes. To achieve this, counts of the KOs that were shown to be differentially abundant between the more restrictive 40% and 60% diets and the 80% diet were extracted. Additionally, KO counts were scaled only by the abundances of the subset of genomes shown to be differentially abundant between the same 40 to 80% and 60 to 80% diet contrasts (30 MAGs in total). All counts were then aggregated into the KEGG BRITE pathway modules for amino acid metabolism, carbohydrate metabolism, energy metabolism and nucleotide metabolism and grouped according to their taxonomic classification (Fig. 10, Table S7). The overall amino acid metabolism, carbohydrate metabolism and energy metabolism all show a greater change in abundance to the 80% diet when comparing the 60% and 40% diets (Fig. 9), whereas KOs associated with the nucleotide metabolism pathway show no overall change when comparing either the 40% or 60% diets to the 80% MER diet. The change in total contribution of differentially abundant MAGs to metabolic pathway modules between 40% and 80% and 60% and 80% diets is increased for amino acid metabolism, carbohydrate metabolism, energy metabolism and nucleotide metabolism, and this increase is greater when comparing 60 to 80% than when comparing 40 to 80% (Fig. 10). The contribution of individual taxonomic groups is more varied, with genomes identified as *Prevotella* and *Clostridiales* contributing an average of 1.56 and 3.11 LFC, respectively, across all pathways in the 40% diet when compared to the 80% diet, but contributing an average of − 3.39 and − 2.46 LFC to all pathways in the 60% vs the 80% diet (Fig. 10, Table S9). Furthermore, despite some small changes in contribution by other taxonomic groups, the larger increase associated with the 60% compared to the 80% diet can almost all be attributed to genomes classified as members of the *Bacteroides* and *Bacteroidales* (Fig. 10, Table S9).

## Discussion

Here, we present 1200 high-quality metagenomic assembled genomes from the rumen of African Zebu exposed to a restricted feeding regime representing the food-scarce circumstances faced by many smallholder farmers in sub-Saharan Africa. Although not a direct estimation of abundance, the distribution of MAGs classified in each of the major rumen phyla, *Bacteroidetes* (29.76%), *Firmicutes* (44.35%) and *Proteobacteria* (7.65%), is comparable to the abundance of these phyla in other cattle rumen communities [23] and those of indicine or cross breed cattle origin, whether using a 16S amplicon sequencing approach [8, 24, 25] or metagenomic read classification [26]. The proportion of MAGs classified to each of the major rumen phyla is also comparable to those recovered from Scottish cattle [11]. There were 50 MAGs (5.88%) in the dereplicated ‘winning’ genome set classified as members of the *Lentisphaerae*, and considering all 1200 genomes that passed filtering, this number rises to 76 (6.33%). Furthermore, estimation of MAG abundances highlighted that, on average, the *Lentisphaerae* constitute 12.5% of the rumen microbiome in these samples. *Lentisphaerae* generally constitute less than 1% of the rumen microbiome, but have been previously shown to be linked to feed efficiency in steers, and in particular in steers with a higher daily gain in bodyweight [25]. This could indicate that their role in the rumen during periods of starvation may be more important when it is required for feed to undergo a greater degree of digestion to maximise energy conversion.

In 16S marker gene studies of microbiomes in general, the move from operational taxonomic unit (OTU)-based methods to amplicon sequence variant (ASV) approaches [27] strives to capture more of the true diversity within a microbial ecosystem. However, the greater resolution provided by whole genome sequencing and further more in MAG reconstruction-based studies will only serve to enhance the understanding of complex microbiomes such as the rumen. Thus, the idea of the rumen ecosystem being subdivided into core and variable communities across localities and host species [8] may only be true in the context of inherently lower resolution 16S marker gene studies. In particular, what is considered the core community more likely represents an underestimation of the true level of diversity in the genomes of rumen organisms. While the African MAGs show taxonomic similarity to taxa commonly found in the rumen, comparison of the genomes using both Mash distance and average nucleotide identity by pairwise alignment indicated that no MAG showed greater than 99% similarity to genomes from the RUGs assembled from Scottish cattle or the Hungate collection. In a 4-Mb genome, this can still represent differences in 40,000 bases between two genomes that have been taxonomically classified as the same species or strain. Rumen bacteria show higher non-synonymous to synonymous polymorphism ratios (pN/pS), representing greater genic diversity, than closely related taxa from other environments [28]. High genic diversity may indicate niche specialisation during the degradation of fibre in the rumen [28]. Comparing the distribution of minimum Mash distance of MAGs vs the Hungate genomes with RUGs vs the Hungate genomes, MAGs show an overall greater minimum distance distribution than RUGs with only 4 MAGs (4/850; 0.05%) showing less than 10% distance from a Hungate genome compared to 54 RUGs (54/913; 6%) and 305 RUG 2.0 (305/4941; 6.17%) showing this level of similarity (Fig. 4). These results indicate that the African MAGs show greater genetic diversity than Scottish RUGs to genomes from the Hungate collection. Comparing MAGs to all other genomes, 96.6% (821/850) show less than 90% similarity, clearly highlighting the level of divergence from public rumen genomes, and the novelty of the data presented here (Figs. 4 and 5a). The distribution of minimum Mash distance within a dataset, i.e. any MAG vs the rest of the MAGs or any RUG vs any other RUG genome, also indicates that there is greater genetic diversity within the MAG dataset than in the RUG, RUG 2.0 or Hungate genomes, suggesting that organisms represented by the MAGs may be under greater selective pressure than organisms from other rumen environments and that they may show a greater degree of niche specialisation [28]. As none of the 1200 high-quality MAGs showed 100% identity to another MAG, we have provided all 1200 genomes as a resource to maximise the genomic information used in future studies. To investigate whether the increase in genetic diversity within the MAGs has an effect on the functional potential of the rumen microbiome, the 850 African MAGs were functionally annotated using the dbCAN database to reveal the full suite of carbohydrate active enzymes associated with each genome. While the representation of the six CAZy modules within the dataset as a whole, and associated with the major rumen phyla such as *Bacteroidetes* and *Firmicutes*, shows similarity in terms of abundance and distribution, the representation of individual CAZy families was more variable, with MAGs containing more CAZy families in total and more unique families than the RUGs. Investigation into the unique families indicated that those present in the RUG dataset are all related to fibre or cell wall carbohydrate breakdown. Similarly, in the MAGs, the majority of the unique CAZy families are related to plant cell wall and structural carbohydrates, with the differences in specificity between the unique families likely to represent difference in forage chemistry in the different diets consumed [29]. Additionally, cluster analyses of the CAZyme abundances highlighted differences in the clusters of enzymes associated with cellulose and hemicellulose and xylans [9]. The overrepresentation of Lentisphaerae within the African rumen MAGs also highlighted the portion of the CAZyome that this phylum contributes to, with an abundance of enzymes involved in the metabolism of certain simple sugars (raffinose, stachyose, maltose), likely to be linked to the use of molasses supplementation within the diet of the African cattle. This phylum also contains a higher abundance of GH129 (α-*N*-acetylgalactosaminidase, EC 3.2.1.49), an enzyme that shows activity against animal mucins. Mucin production and dynamics have been shown to be altered by starvation [30] and become a source of nutrition to dietary-fibre starved gut microbiomes [31, 32].

Estimations of MAG abundance further supported that the broad phylum-level taxonomic representation of organisms within the rumen of African Zebu fits what has been seen previously in 16S analyses, with the *Bacteroidetes*, *Firmicutes* and *Proteobacteria* representing 55.42%, 23.31% and 2.95%, respectively [8, 23]. While the *Proteobacteria* were, on average, higher in the most severely restricted 40% MER diet, 3.41% vs 2.52% and 2.93% in the 60% and 80% MER diet treatments, respectively, differential abundance analysis at the phylum level indicated that this was non-significant. An elevated ratio of *Proteobacteria* to *Bacteroidetes* + *Firmicutes*, greater than 1.9, has been shown to represent a dysbiosis in the rumen [33]; however, the levels here fall within the normal ranges seen within the rumen [8, 23]. Further analyses at the MAG level highlighted the same 30 MAGs were significantly differentially abundant (adjusted *P* value < 0.05) when comparing the more restrictive 40% and 60% diets to the higher 80% diet treatment. Comparing the abundances between diet treatments at the MAG level highlights differentially abundant MAGs that are the main drivers underlying discrimination between diets in PCoA ordination analysis (Table S6).

Analysis of the full, overall functional potential of the rumen microbiome and its response to diet restriction showed that overall there are an equal number of MAGs significantly differentially abundant but more with a positive differential abundance (LFC > 0) between 40% and 80% MER diets than 60% and 80% MER diets, with 20 and 16 MAGs, respectively. Similarly, looking at the effect of diet at KO level, i.e. based on the total set of KOs present in all MAGs across all samples, again the total number of differentially abundant KOs when comparing the 40 to 80% diet is the same as when contrasting the 60% and 80% (147), but that a greater number show positive differential abundance (LFC > 0) in the 40% vs 80% than the 60% vs 80% contrast, with 91 and 78, respectively. These differentially abundant sets of KOs represent the overall change in functional potential between the more severely restricted diets and the 80% diet. Furthermore, out of the 7469 pathway modules associated with KOs identified within the MAGs, 2847 (38.1%) are related to metabolic pathways. Within the 147 KOs differentially abundant between the 40% and 80% diets, 54 (36.7%) are associated with metabolic function.

To investigate whether the differentially abundant MAGs are the main drivers of the overall change in functional potential in the rumen, we looked at the functions attributable to the subset of differentially abundant MAGs and compared it with the overall change in functional potential identified in the KO-level analysis (i.e. 147 KOs for 40% vs 80% and 60% vs 80%). By doing so, we highlight three different scenarios when focusing on metabolic pathways.

A first scenario consists in an agreement between the overall change in representation of KOs associated with a given pathway and the functional change attributable to differentially abundant MAGs for that same pathway. For example, the differential abundance of KOs from the amino acid metabolism pathway module is significantly more represented in the metagenomes associated with the 60% MER diet than those on the 80% (Fig. 9; LFC 0.72). If we consider the KOs encoded by the MAGs showing differential abundance between those same diets, there is also an increase (LFC 1.67) in abundance of KOs associated with amino acid metabolism (Fig. 10, Table S9). Looking at the taxonomic classification of the differentially abundant MAGs, 2 were identified as members of the *Ruminococcus*, 4 as *Clostridium* and 1 as unknown member of the *Bacteroides*. The respective contribution of these genomes represents a 3.02 LFC, 3.04 LFC and 7.78 LFC increase in abundance of KOs associated with amino acid metabolism. In contrast, contribution from the 5 *Clostridiales* and 3 *Prevotella* genomes decreases by a − 2.43 LFC and − 3.39 LFC, respectively (Fig. 10, Table S9). While the total effect from the differentially abundant MAGs is in agreement with the overall observed functional change, these findings highlight that not all taxonomic groups within the differentially abundant MAGs contribute to the LFC of 1.67 increase in abundance of KOs associated with amino acid metabolism, but nearly all of the total increase associated with the differentially abundant MAGs can be attributed to the 1 genome identified as unknown member of the *Bacteroides* (Fig. 10, Table S9).

The second scenario highlights a disconnect between overall change in functional potential and the change attributable to the significantly differentially abundant MAGs. For example, when comparing the metagenomes associated with the 40% diet to that of the 80% diet, we see small negative changes in representation of the carbohydrate metabolism (LFC of − 0.13) pathway (Fig. 9), but larger, positive changes in this pathway when considering only the 30 significantly differentially abundant MAGs, with LFC of 1.34, respectively (Fig. 10, Table S9). Looking further at the contribution of individual taxa to the pathways, positive changes are seen in the contribution of *Ruminococcus*, *Clostridium*, *Clostridiales*, *Prevotella* and *Bacteroides* genomes to the carbohydrate metabolism pathway (LFCs of 4.89, 1.21, 3.07, 1.61 and 1.09, respectively). As observed in the first scenario, not all taxonomic groups within the differentially abundant MAGs contribute to the increase in abundance of KOs associated with this metabolic pathway.

Finally, the third example highlights a scenario where no significant change can be seen in the overall functional potential, but significant change would be concluded when considering only the differentially abundant MAGs. When comparing the metagenomes associated with the 40% and 60% diet to that of the 80% diet, no significant change is seen in any KO associated with nucleotide metabolism (Fig. 9). However, according to the change attributable to the differentially abundant MAGs, with a LFC of 1.07 for the 40% vs 80% diet and 1.16 for the 60% vs 80% diet, this pathway shows greater representation in the lower MER diet samples (Fig. 10, Table S9). In the 40% vs 80% diet contrast, the largest contribution comes from the 2 genomes identified as *Ruminococcus* with a LFC of 5.02, whereas in the 60% vs 80% contrast, the largest contributor of change in this pathway is the 1 *Bacteroides* genome (LFC of 7.78) (Fig. 10, Table S9).

These three scenarios highlight that significant observations of change in rumen functionality may not always be associated with a large change in an individual taxon or group of taxa, and could instead be associated with a shift in the rumen microbiome as a whole, made up of many small-scale differences, considered insignificant on their own.

In the study of the rumen, the more common 16S marker gene-based studies are inherently limited by their lack of functional data, and although steps have been taken to use tools to infer functional potential [10, 34], this genome-centric MAG-based methodology provides a more direct connection between taxa and function. Analysis based on MAG abundance also provides a further level of resolution than previous metagenome-wide functional analyses, where changes in function may be seen, but it is then difficult to associate them with your taxa of interest, as the change could be a result of a shift in the entire rumen microbiome. As such, any future effort in targeting interventions to impact ruminant production and efficiency should take careful consideration of both whole-rumen function and taxon-level changes in community structure. We suggest that use of MAG discovery and abundance-based analyses currently represents a key tool to investigate the responses of the rumen microbiome and its involvement in feed efficiency in ruminant production.

## Conclusions

We characterised the rumen microbial composition of Boran cattle living in sub-Saharan Africa. This indigenous cattle breed is known to be well adapted to harsh tropical environments with low-quality food and feed scarcity. The de novo assembly of metagenome-assembled genomes revealed 1200 high-quality African rumen-specific MAGs, 850 of which were unique on the basis of 99% ANI. Furthermore, when we investigated diet restriction in indigenous African cattle and its effect on rumen-wide shifts in community structure and functional potential, the extra resolution provided by MAG-based analysis highlighted three distinct scenarios, two of which show a disconnect between significant taxonomic changes and changes in the overall rumen functional potential. Such observations cannot be made with 16S marker gene analysis and have been overlooked in previous metagenomic studies. These novel MAGs represent a fundamental resource that further helps in deciphering the rumen microbiome and its contribution to functionality, efficiency and production in indigenous Zebu cattle in an African smallholder-specific context.

As demand for animal products in this region increases, so too does the necessity for efficiency in the livestock production chain. The rumen microbiome has long been studied in the context of improving animal production through fibre degradability for feed efficiency or nutrient availability and utilisation and its effects on meat and dairy products. Despite this, these studies often only address the challenges in the context of the commercial farm. Rural, smallholder farms in Africa hold the key to providing local-scale food security, and investigation into the improvement of these systems will protect these vital resources as demand increases. The African MAG data will also provide a vital resource in future studies looking to manipulate the rumen to increase efficiency and production in the unique system of rural farming in Africa with the goal of addressing food security in developing countries.

Additionally, the development of metagenomic-driven culture studies, the so-called culturomics, will add to the study of microbiomes as a whole [35], and MAGs will provide an invaluable ‘stepping stone’ to the cultivation, isolation and sequencing of previously unculturable taxa [36].

## Methods

### Sample collection and sequencing

All animal procedures were carried out adhering to international standards for animal care and use for scientific purposes, reviewed by the Institutional Animal Use and Care Committee of the International Livestock Research Institute (ILRI) permit no: IACUC-RC2016-11.

Six Boran yearling steers, matched for age and live weight, were sourced from a commercial ranch in Lakipia Province (Nth Kenya). Cattle were fed three sub-optimal diet treatments, i.e. 40%, 60% and 80% maintenance energy requirements (MER), as well as a 100% diet treatment, over 4 periods in a crossover design (Table 7). Experimental diets were based on an allocation chaffed Rhodes grass (*Chloris gayana* cv. Boma DM; 875 g/kg; DE:8.4 MJ/kgDM; CP:73.1 g/kgDM) late-cut hay, plus the addition of a small amount of cottonseed meal (DM; 947 g/kg; DE:12.7 MJ/kgDM; CP:324.4 g/kgDM) and molasses (DM; 728 g/kg; DE:14.2 MJ/kgDM; CP:46 g/kgDM) to the ration of animals being fed at 100% MER, in order to achieve required intake, a process recommended to African smallholder farmers [37] and common in East Africa [38, 39]; with DM, DE and CP referring to dry matter, digestible energy, and crude protein respectively. The experimental design and diet treatments used are based on the study by Goopy et al. (personal communication). Briefly, treatment periods outlined in Table 7 were 35 days in length, including 21 days of adaptation. Prior to each treatment period, animals underwent a 14-day feed-up period, fed at 100% MER to minimise carry-over effect of the treatment period. Rumen digesta was sampled by stomach tubing from each animal during the fourth week of each period of the trial at the same time as other measurements were taken [40, 41], for a total of 24 samples.

**Table 7:** Experimental design, animals were fed 4 diet treatments over 4 periods in a crossover design

Animal ID	% MER diet			
	Period 1	Period 2	Period 3	Period 4
41	80	60	40	100
43	60	40	100	80
46	100	80	60	40
47	40	100	80	60
49	40	100	80	60
52	100	80	60	40

Based on live weight determined at the beginning of each period, 6 animals were fed at 40%, 60%, 80% or 100% of their maintenance energy requirements (MER) over 4 periods in a crossover design. Treatment periods were 35 days and were preceded by a 14-day feed-up (100% MER) to minimise any carry-over effect. Sampling was carried out in the fourth week of each treatment period

Samples taken during period 1 were frozen until required for DNA isolation, and samples from periods 2–4 were stored frozen in 50% glycerol until required for DNA isolation. Previous studies have shown that while inclusion of a cryoprotectant does not affect the presence/absence of rumen micro-organisms detected in a sample, minor effects on abundance of taxonomic groups have been observed [42, 43]. Therefore, all 24 samples were processed through the MAG discovery pipeline outlined in Fig. 1, and to reduce bias in the abundance calculations, samples from period 1 were not included in the downstream analysis investigating the impact of diet restriction. As has been shown previously, diet is the main driver of microbial change within the rumen [8]. The 100% MER diet consisted of additional cottonseed and molasses, which represents a different dietary composition and carbohydrate profile of this treatment. Due to the confounding nature of this compositional difference with our investigation into the effect of dietary amount through % MER treatments, samples associated with the 100% MER diet were not included in downstream taxonomic and functional comparisons.

DNA extraction was carried out following the protocol of Yu and Morrison [44] and based on repeated bead beating plus column filtration. Illumina TruSeq libraries were prepared from genomic DNA and sequenced on an Illumina HiSeq 4000 by Edinburgh Genomics.

### MAG assembly

In order to reconstruct metagenome-assembled genomes (MAGs) from all samples, we followed the bioinformatics workflow as shown in Fig. 1, and described below.

Adapters were trimmed from the Illumina data using Trimmomatic [15] and the subsequent trimmed reads used as input for MEGAHIT [17]. A 24-metagenome co-assembly was carried out using options --kmin-1pass, -m 60e + 10, --k-list 21,31,41,51,61,71,81, --min-contig-len 300, -t 16. In addition, 24 single-sample assemblies were performed using IDBA-UD [16] with the options --num_threads 16 --pre_correction --min_contig 300. BowTie2 [45] was used to map reads back to the filtered assembly, and SAMtools [46] was used to convert to BAM format. Script jgi_summarize_bam_contig_depths from the MetaBAT2 [47] package was used to calculate coverage from the resulting BAM files.

Metagenomic binning was applied to both single-sample assemblies and the co-assembly using MetaBAT2 [47], with options --minContigLength 2000, --minContigDepth 2. Coverage values across the 24-sample dataset were considered.

To determine whether the same genome has been reconstructed via the different assembly approaches, all bins were aggregated and then dereplicated using dRep [19]. The dRep dereplication workflow was used with options dereplicate_wf -p 16 -comp 80 -con 10 -str 100. dRep utilises CheckM [18] to first remove genomes with a completeness score of less than 80% and contamination higher than 10%. These high-quality MAGs are then subjected to fast pairwise comparison via a Mash [21] algorithm and grouped into primary clusters at 90% average nucleotide identity (ANI). Members of each primary cluster then undergo more stringent pairwise comparison to group MAGs into secondary clusters at 99% ANI using ANIm algorithm [48] in combination with MUMmer (V 3.0 [49]). Genomes are scored on the basis of completeness, contamination, genome size and contig N50, with only the highest scoring MAG from each secondary cluster being retained as the winning genome in the dereplicated set.

### Taxonomic classification of MAGs

For taxonomic classification of the 850 dereplicated MAGs, the Snakemake pipeline MAGpy was employed [20]. This pipeline utilises a number of underlying tools to attempt to taxonomically classify MAGs based on a set of core genes (via CheckM) or protein families (via DIAMOND BLAST and PhyloPhlAn). Pearson’s and Spearman’s rank correlations were calculated for counts of genomes classified as members of each rumen phylum to classifications of the original Scottish RUG and RUG 2.0 genomes.

### Uniqueness/divergence

To assess uniqueness to the rumen microbiome of African cattle, the 850 MAGs were then further dereplicated with the 913 rumen MAGs produced by Stewart et al. [11] from Scottish cattle. At 90% ANI, only 21 of the primary clusters in the dRep workflow contained both an African MAG and a Scottish RUG. Secondary pairwise comparison at 99% average nucleotide identity indicated no overlap in African MAGs and Scottish RUGs, with all 850 African rumen MAGs being retained in the winning set. To further investigate the genetic divergence of the African MAGs from other publicly available genomes of rumen organisms, MAGs were further compared to the original 913 Scottish RUGs, the 4941 RUG 2.0 [12] and the 460 publicly available genomes from the Hungate collection [9], using Mash to calculate Jaccard distances for every pairwise comparison of the rumen genomes, based on number of shared 21-mers out of a possible total of 100,000. The minimum distance for each genome to any genome from one of the other datasets was plotted to represent the distribution of genetic divergence (Fig. 4).

### Functional classification of MAGs

As the greater level of genetic diversity shown in the African MAGs is not a function of greater taxonomic diversity, it is likely to impact the functional genetic content of the genomes. To assess the contribution of the constructed MAGs to the functional potential of the rumen microbiome, the 1,613,102 predicted proteins extracted by Prodigal during the CheckM pipeline were compared to the KEGG database using DIAMOND BLAST; hits were filtered to only those with a minimum identity of 50% and an *E* value of less than 1e−5; resulting KEGG orthologs (KOs) were further compared to the KEGG BRITE database to identify involvement in functional pathways. Additionally, annotation of carbohydrate active enzyme (CAZymes) in the MAG proteins was performed using dbCAN [22] database, a collection of HMM profiles built based on the carbohydrate active enzyme (CAZy) database. Hits were filtered for a minimum coverage of 35% and an *E* value less than 1e−18.

To assess the novelty, the 68,850 filtered proteins identified as having carbohydrate activity were compared to the nr, env_nr, M5nr [50] and UniProt/TrEMBL [51] databases. The proteins were also compared to the predicted proteins from the Hess [52] dataset and the predicted proteins from the original Scottish RUGs [11] and RUG 2.0 [12] genomes.

To investigate substrate specificity of carbohydrate degrading taxa, heatmaps representing log counts of enzymes belonging to each CAZy family, in each MAG, were constructed using pheatmap package (V1.0.10, [53]). Hierarchical clustering was performed with Spearman’s rank correlation as distance and pairwise average-linkage as clustering method. Columns were clustered by unpaired group means of Spearman’s correlation, and rows were arranged taxonomically [9].

### Effect of diet

To investigate the effect of the different sub-optimal diet treatments (80%, 60% and 40% MER) on the rumen microbiome, the abundance of each MAG within each sample was estimated. The original, trimmed reads were mapped to each contig associated with each MAG using BowTie2 [45]. Reads contributing to each contig were counted on a MAG per sample basis using SAMtools [46] to create a contig coverage matrix for all samples. Using the Bioconductor [54] package EDAseq [55], coverage estimates were corrected for contig length and GC content bias, to produce a MAG count per sample matrix. This count matrix, the metadata (Table S10) and taxonomy table (formatted to retain taxonomic information across ranks; Table S11) were used as input to the Bioconductor package phyloseq [56]. For all population and community analyses (PERMANOVA, PCoA), the MAG count per sample matrix was also Hellinger transformed and batch corrected for animal and period effects using the R package limma [57, 58], and the Euclidean distances between samples calculated [59]. The Hellinger transformation represents the square root of the relative abundance. This transformation accounts for variations in library size and for the sparse matrices associated with microbiome studies. The Adonis2 function in the vegan package [60] was used to perform PERMANOVA on the transformed and batch corrected data, to analyse difference in community structure between sample groups using 9999 permutations and corrected for multiple testing using the Benjamini-Hochberg [61] method. Principal coordinate analysis (PCoA) analyses were performed using Euclidean distance matrices.

To investigate differential abundance of taxa between diet treatments, the Bioconductor package DESeq2 [62] was used in R (with the samples grouped by diet, and the more restrictive 60% and 40% diets compared to the 80%). Here, the MAG count matrix that was corrected for GC content and contig length using EDAseq (Table S5) was used (with no Hellinger transformation, and no batch effect correction), as the DESeq2 model internally corrects for library size [63], along with metadata and taxonomy tables (Tables S6 and S7). A full model design including period, animal and diet effects (~ Period + Animal + Diet) was tested against a reduced model including only period and animal effects (~ Period + Animal) using the likelihood ratio test (LRT) in DESeq2, to determine taxa that show significant differential abundance between the diet treatments. An R script (Additional file 15) is included to perform the DESeq2 analysis as described above.

To assess the effect of diet on the functional potential of the rumen microbiome of African cattle, the KO counts per MAG (Table S3) were multiplied by the abundance of each MAG per sample (Table S5) to generate a KO per sample matrix (Table S7) to represent the functional potential of the rumen microbiome of each sample. The resulting count matrix representing functional potential was then used as input, along with a metadata table (Table S10) for the Bioconductor package DESeq2 [62] in R (with the samples grouped by diet, and the more restrictive 60% and 40% diets compared to the 80%), and assessed using LRT with the same design (full model vs reduced model) as mentioned previously (analysis also included in Additional file 15). The KEGG BRITE pathway modules, amino acid metabolism, carbohydrate metabolism, energy metabolism and nucleotide metabolism were selected for highlight and visual representation of differential abundances as of all of the metabolism-associated modules these are made up of the highest number of representative KOs.

#### Power calculation analyses

Power calculation analyses use the relationship between power, sample size, effect size and significance value (*P* value). PERMANOVA analyses (using Adonis2 from the R package vegan) included in the ‘Effect of diet restriction on rumen microbiome structure and functional potential’ section used the diet as the grouping factor to test significance. The experimental design of 4, 5 and 5 samples representing 40%, 60% and 80% MER (4:5:5 experimental design), respectively, was associated with effect sizes of 44.6% (R2 = 0.446) and 45.9% (R2 = 0.459) for MAG and KO relative abundance data, respectively. Using the R package micropower [64], power calculation analyses were conducted to assess the variation in statistical power in different experimental designs. Using a random sampling with replacement approach on the Hellinger transformed count matrices that had been batch corrected for animal and period effects, we generated 1000 Euclidean distance matrices for a range of sample size groupings (3:3:3, 4:4:4, 4:5:5 and 5:5:5 samples), selected to investigate variation in statistical power with groupings including both lower and higher sample sizes per treatment. The bootstrapped PERMANOVA power function within micropower estimated statistical power for each matrix based on 10,000 bootstrap permutations, for a total of 10 million estimates for each sample size grouping. This analysis was conducted for *P* values of 0.05, 0.01 and 0.001 and indicated that even at 0.001 (highly significant threshold), the median power across 10 million permutations was 0.99 for the 4:5:5 design and 0.99 for the 5:5:5, but only 0.84 for the 4:4:4 design and no statistical power for the 3:3:3 design (0.03). Analyses at *P* values of 0.05 and 0.01 indicated powers of 1 for the 4:5:5 and 5:5:5 designs at both significance *P* values (Fig. S3). These results clearly indicate a strong statistical power (0.99 at a *P* value 0.001) associated with the sample size groupings (4:5:5) in the experimental design used in this study.

#### Interplay of significantly differential abundant genomes with significant change in functional potential

To highlight the power of our genome-centric approach in the analysis of the rumen metagenome, significant changes in the overall functional potential of the microbiome were contrasted with differences accounted for by the significantly differentially abundant genomes. To achieve this, counts of the KOs that were shown to be differentially abundant between the more restrictive 40% and 60% diets and the 80% diet were extracted. Additionally, KO counts were scaled only by the abundances of the subset of genomes shown to be differentially abundant between the same 40 to 80% and 60 to 80% diet contrasts (30 MAGs in total). All counts were then aggregated into the KEGG BRITE pathway modules for amino acid metabolism, carbohydrate metabolism, energy metabolism and nucleotide metabolism and grouped according to their taxonomic classification (Fig. 10, Table S9).

## Supplementary information

**Additional file 1: Fig. S1.** Heatmaps displaying counts of enzymes belonging to each CAZyme family, in each MAG. Clusters of CAZyme families involved in the breakdown of selected polysaccharides (highlighted by coloured boxes). Heatmaps generated from CAZymes present in African MAGs. Figure 7a in high resolution.

**Additional file 2: Fig. S2.** Heatmaps displaying counts of enzymes belonging to each CAZyme family, in each MAG. Clusters of CAZyme families involved in the breakdown of selected polysaccharides (highlighted by coloured boxes). Heatmaps generated from CAZymes present in Scottish RUGs. Figure 7b in high resolution.

**Additional file 3: Fig. S3.** Distribution of estimated statistical power. Sampling with replacement generated 1000 Euclidean distance matrices based on our MAG abundance data for a range of sample size groupings (3:3:3, 4:4:4, 4:5:5 and 5:5:5) representing 40%:60%:80% MER diet treatments respectively. The bootstrap PERMANOVA function estimated statistical power for each matrix based on 10,000 bootstrap permutations. Distribution of the power estimates is shown for each experimental design and at *P* = 0.05 (panel A), *P* = 0.01 (panel B) and *P* = 0.001 (panel C).

**Additional file 4: Table S1.** Taxonomic classifications of 850 winning African MAGs. Taxonomic classifications reported for each of the three following methods: CheckM, DIAMOND and PhyloPhlAn.

**Additional file 5: Table S2.** Minimum Jaccard distances. Minimum Jaccard distance between all genomes within a single dataset from either African MAGs (MAG), Scottish RUGs (RUG1), RUG 2.0 (RUG2) or Hungate collection (HUN); between two datasets (MAGvRUG1, MAGvRUG2, MAGvHUN, RUG1vRUG2, RUG1vHUN, RUG1vMAG, HUNvRUG1, HUNvRUG2, HUNvMAG, RUG2vRUG1, RUG2vHUN, RUG2vMAG); between a given dataset and all other datasets combined (MAGvAll, RUGvAll, RUG2vAll, HUNvAll).

**Additional file 6: Table S3.** KEGG Functional Annotations of MAGs. Counts of proteins in each MAG with a hit in the KEGG database for a KEGG ortholog (KO_Counts).

**Additional file 7: Table S4.** CAZyme Functional Annotations of MAGs. Counts of proteins in each MAG with a hit in the dbCAN CAZyme database (CAZy_Counts).

**Additional file 8: Table S5.** Matrix of read counts per MAG. Read counts per MAG were calculated based on reads mapped to all contigs of a MAG, corrected for contig length and GC content bias. Used as input for phyloseq and DESeq2 analyses.

**Additional file 9: Table S6.** Significantly differentially abundant MAGs. Significantly differentially abundant MAGs comparing 40% (40vs80) and 60% (60vs80) MER diet treatments versus the 80% treatment. Differential abundance was calculated using the likelihood ratio test (LRT) in DESeq2 and was considered statistically significant at FDR (adjusted *p*-value) < 0.05 threshold.

**Additional file 10: Table S7.** Matrix of KO counts per Sample. KO counts per MAG (Table S3) were multiplied by the abundance of each MAG per sample (Table S5) to generate a KO per sample matrix representing the functional potential of the microbiome associated with each sample to be used as input for phyloseq and DESeq2 analyses.

**Additional file 11: Table S8.** Overall significantly differentially abundant KOs. Significantly differentially abundant KOs associated with metabolically important pathways in the rumen when comparing 40% (40vs80) and 60% (60vs80) MER diet treatments versus the 80% treatment. Differential abundance was calculated using the likelihood ratio test (LRT) in DESeq2 and was considered statistically significant at FDR (adjusted p-value) < 0.05 threshold.

**Additional file 12: Table S9.** Contribution of differentially abundant MAGs to metabolic pathways. Absolute counts of KOs associated with significantly differentially abundant MAGs comparing 40% and 60% MER diet treatments versus the 80% treatment. Counts for each taxonomic group in each diet treatment are aggregated into metabolically important pathways within the rumen (KO counts). Using the absolute counts, the log 2 fold change (LFC) of the counts in the 40% and 60% diet treatments has been calculated to give proportional change in contribution of each taxonomic group to each metabolic pathway when compared to the 80% diet treatment (LFC vs 80%).

**Additional file 13: Table S10.** Metadata Table for Metagenome Samples. Metadata table containing Sample ID, Period, Animal and Diet treatment information for each sample adapted from Table 7 to be used as input for phyloseq and DESeq2 analyses.

**Additional file 14: Table S11.** Taxonomy Table of MAGs. Complete taxonomy table adapted from Table S1 to be used as input for phyloseq and DESeq2 analyses.

**Additional file 15:** R Script for DESeq2 and phyloseq analyses. R script to perform MAG and KO level using the likelihood ratio test (LRT) in DESeq2. Analyses identify significantly differentially abundant MAGs and KOs when the more restrictive 40% and 60% MER diet treatments are contrasted against the 80% MER diet treatment. Statistical analysis of the ruminal community structure is performed using the Adonis2 PERMANOVA analysis. Principal coordinate analysis (PCoA) and canonical analysis of principal coordinates (CAP) ordination plots are generated using phyloseq and ggplot2.

**Additional file 16:** Review history.

## Data Availability

Sequence data has been deposited in the European Nucleotide Archive under the project accession PRJEB39057 [65]. All raw reads and metagenome-assembled genomes will be released under this project.
