# Supplementary figures and images for: 1200 high-quality metagenome-assembled genomes from the rumen of African cattle and their relevance in the context of sub-optimal feeding
Source: Genome Biol. 2020 Sep 3;21:229. doi: 10.1186/s13059-020-02144-7 (PMC7469290; doi:10.1186/s13059-020-02144-7)

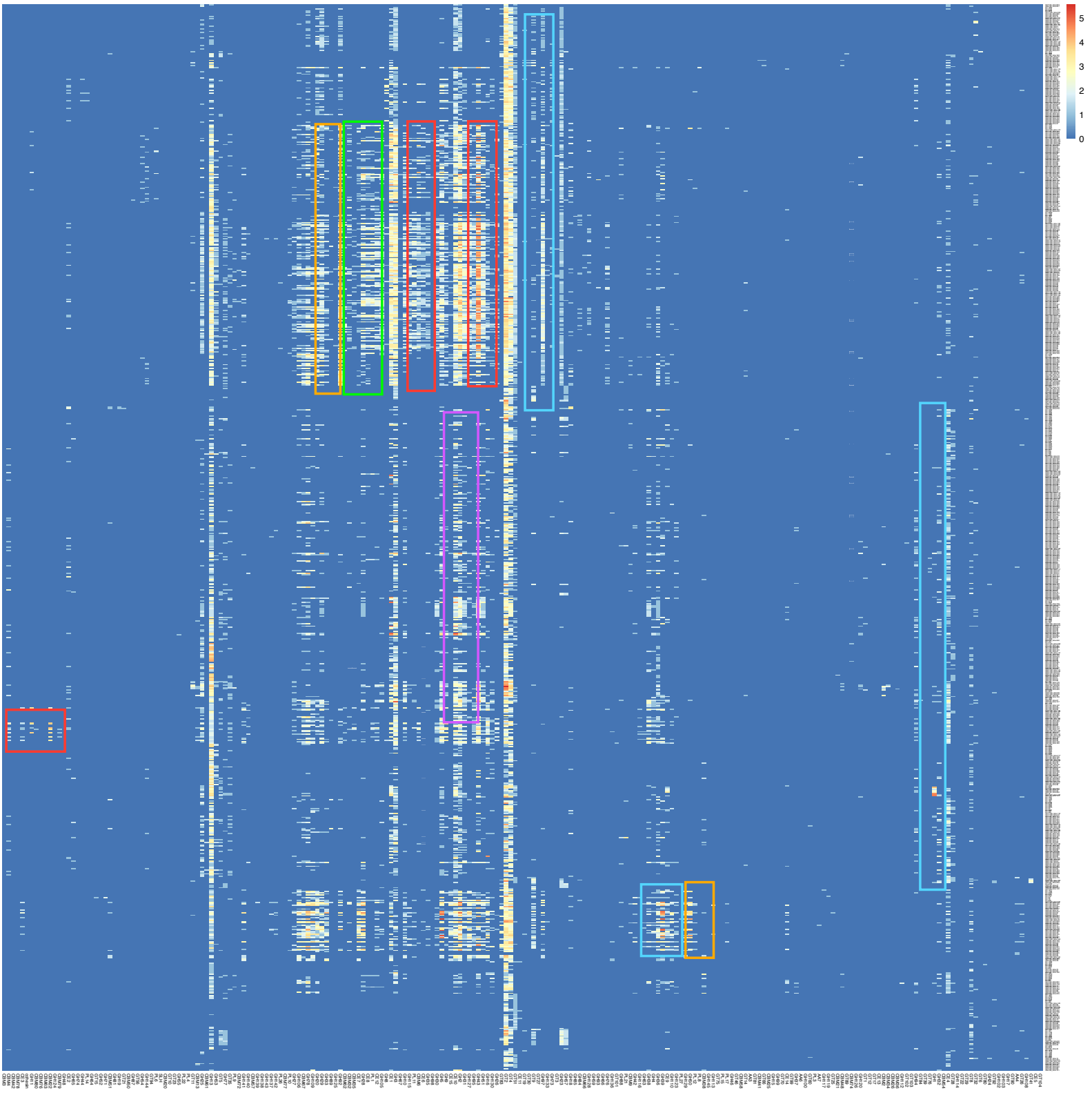

Supplement: Supplementary file 1 — Additional file 1: Fig. S1. Heatmaps displaying counts of enzymes belonging to each CAZyme family, in each MAG. Clusters of CAZyme families involved in the breakdown of selected polysaccharides (highlighted by coloured boxes). Heatmaps generated from CAZymes present in African MAGs. Figure 7a in high resolution. [file 13059_2020_2144_MOESM1_ESM.pdf]

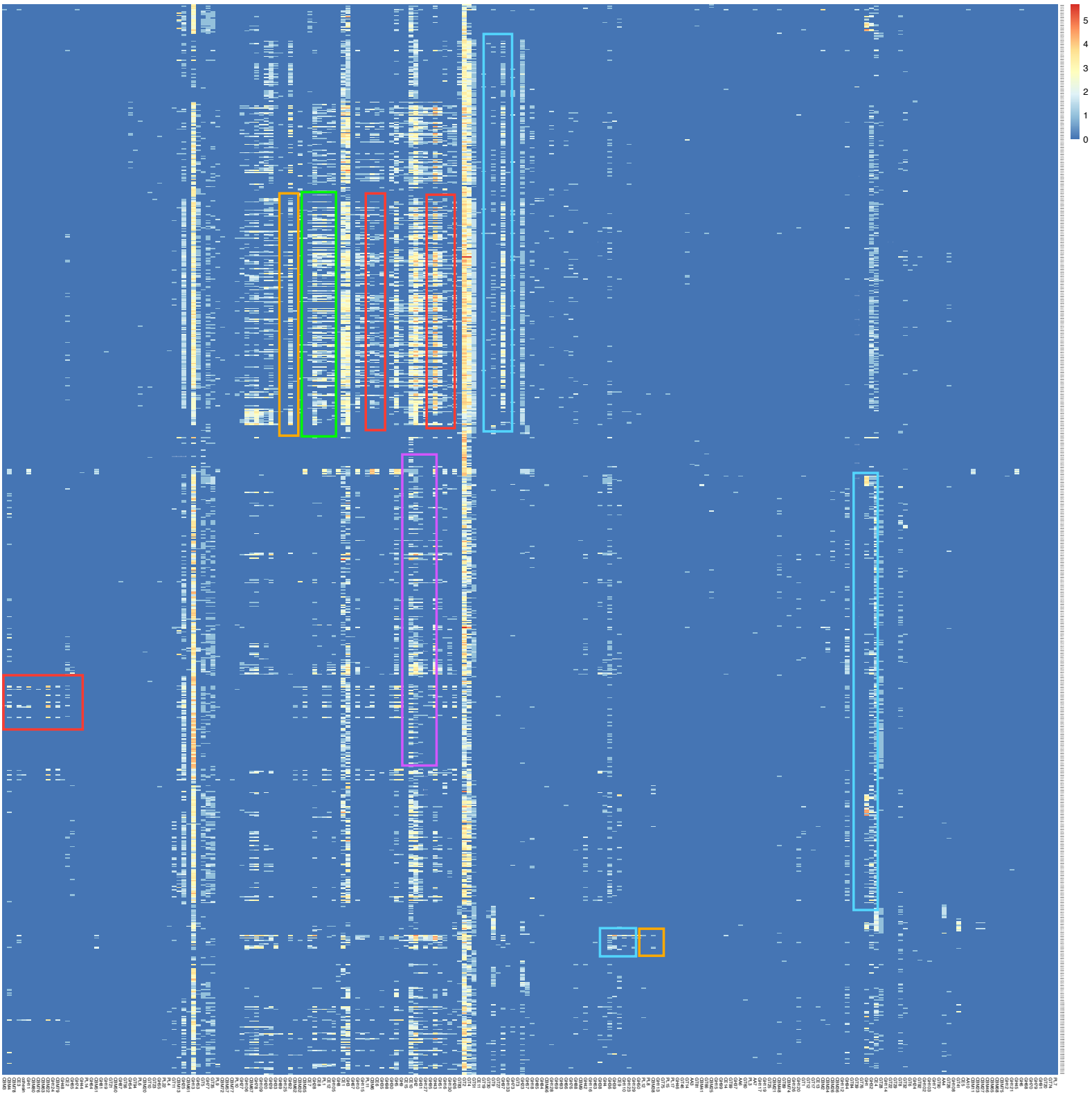

Supplement: Supplementary file 2 — Additional file 2: Fig. S2. Heatmaps displaying counts of enzymes belonging to each CAZyme family, in each MAG. Clusters of CAZyme families involved in the breakdown of selected polysaccharides (highlighted by coloured boxes). Heatmaps generated from CAZymes present in Scottish RUGs. Figure 7b in high resolution. [file 13059_2020_2144_MOESM2_ESM.pdf]

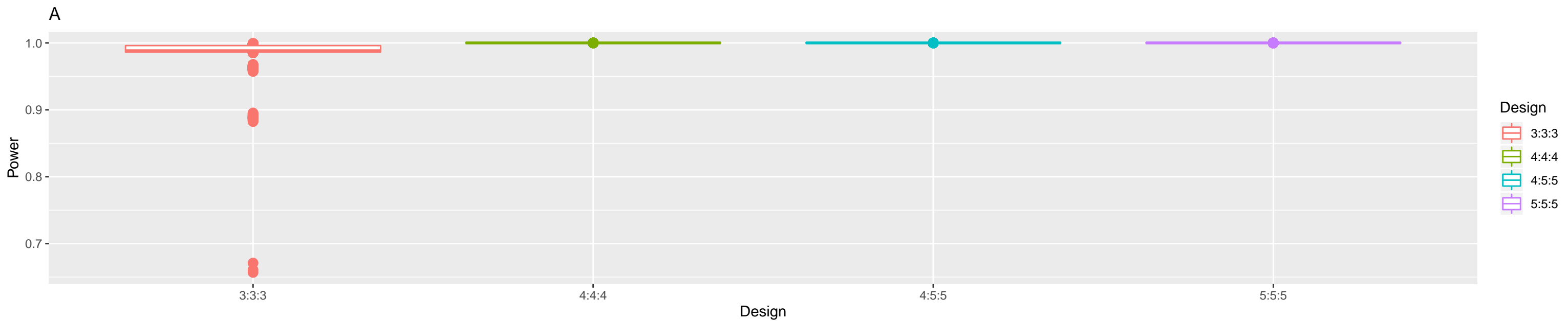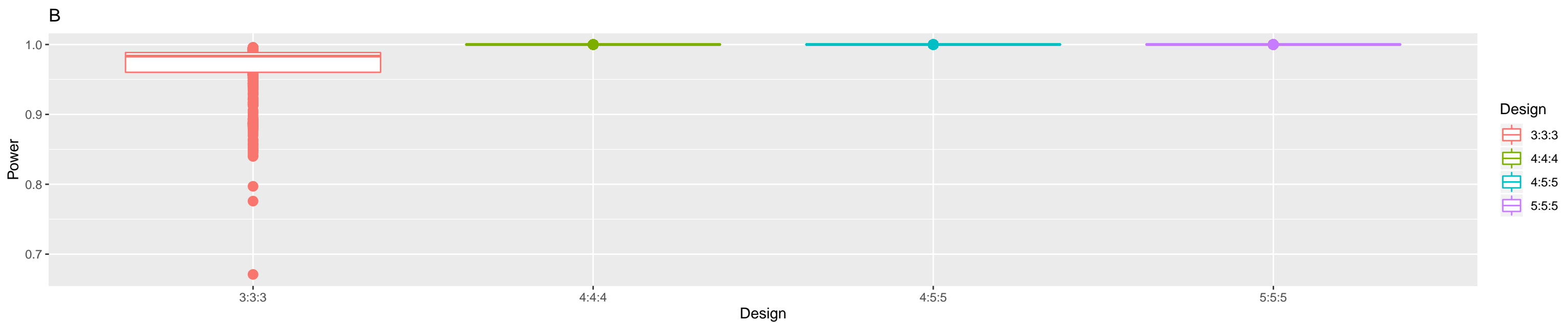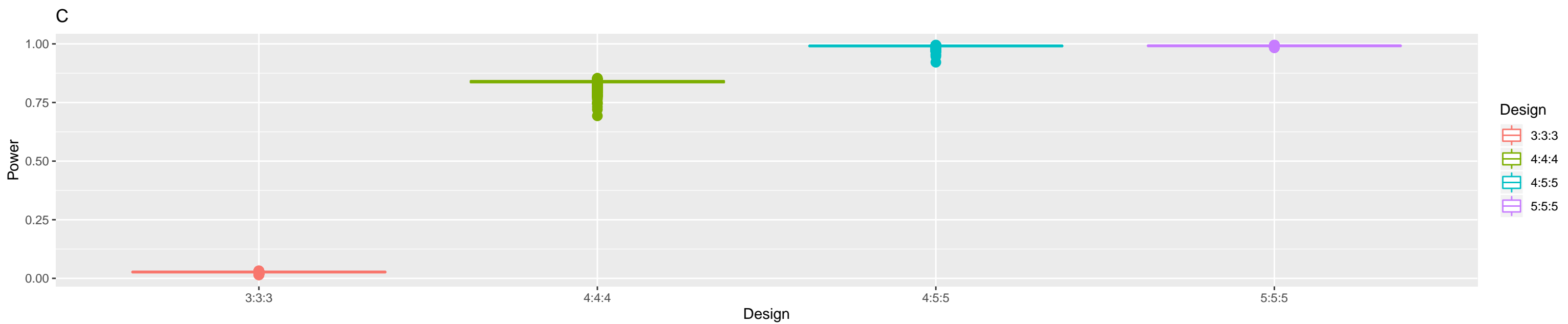

Supplement: Supplementary file 3 — Additional file 3: Fig. S3. Distribution of estimated statistical power. Sampling with replacement generated 1000 Euclidean distance matrices based on our MAG abundance data for a range of sample size groupings (3:3:3, 4:4:4, 4:5:5 and 5:5:5) representing 40%:60%:80% MER diet treatments respectively. The bootstrap PERMANOVA function estimated statistical power for each matrix based on 10,000 bootstrap permutations. Distribution of the power estimates is shown for each experimental design and at P = 0.05 (panel A), P = 0.01 (panel B) and P = 0.001 (panel C). [file 13059_2020_2144_MOESM3_ESM.pdf]
