# Supplementary material for: 1200 high-quality metagenome-assembled genomes from the rumen of African cattle and their relevance in the context of sub-optimal feeding
Source: Genome Biol. 2020 Sep 3;21:229. doi: 10.1186/s13059-020-02144-7 (PMC7469290; doi:10.1186/s13059-020-02144-7)
Supplement: Supplementary file 16 — Additional file 16: Review history. [file 13059_2020_2144_MOESM16_ESM.docx]

**1^st^ round**

**Reviewer 1**

This manuscript reported 1200 metagenome assembled genomes (MAGs) from the rumen of Zebu cattle under different dietary conditions. Although the identified MAGs do add more information to the current data pool of rumen microbiome, the current version is not suitable for being published at Genome Biology. The main issues are:

1. The concept is not new and novel. The work flow and contents are pretty similar to Stewart et al.(2018) except using rumen samples generated from different cattle.

2. Lack of fundamental biological questions and biological relevant interpretation of the results.

a. The differential abundant ones had very low relative abundance. As described above, the manuscript does not have any new unanswered questions in terms of rumen microbiome.

b. It seems strange that the identified MAGs could not be found in known databases. It is very doubtful that the rumen microbiome of zebu cattle is completely different from those in other ruminants. Based on tremendous published 16S data and metagenome dataset, the difference between rumen microbiota of Bos Taurus and Bos Indicus do share many taxa. It is important to verify whether the identified novel MAGs are not just artifacts of computational based analysis.

c. The identified significant differential abundant MAGs in response to diet are all extremely low abundant ones (less than 0.05%, Table 3). It is quite common for those low abundant ones to be significant different. Are these MAGs biological meaningful? Also, why the comparison is based on phylum level not lower taxonomic level?

3. The sample size is small with only 6 cattle comparing to 43 in Stewart et al.(2018). The difference in abundances of MAGs under three dietary conditions were also just based on 6 cattle, which has very little power. Also, not clear why only three dietary conditions were compared, not four?

4. Lack of descriptive information of sample sources which makes the results less convincing.

Based on Stewart et al.(2018), they generated 850 MAGs from 768Gb data. It is unclear how much sequence data were generated, if it is only 6 cattle with 106 million reads, the total amount of data was only 106MX150X6/1024M=93.16Gb. And how can such low about of data could generate more MAGs than Stewart et al.(2018)?

5. Lack of validation: All conclusions are based on bioinformatic analysis. For high impact journal like Genome Biology, the discovery in biological process is essential. There are some fundamental questions need to be answered through following up experiment. For example,

a. Are all MAGs true colonizers? And do they only colonize in zebu? Are those novel MAGs having unique metabolic pathways and/or functions (such as detoxification) comparing to all known rumen cultures and MAGs? If so, will they be able to degrade different type of forages, for example, the forages only grown in Africa?

b. CAZymes: are there any of them novel enzymes? If so, can authors verify that they are actually produced in the rumen?

**Reviewer 2**

This paper presents 1200 high quality MAGs from 6 Zebu cattle, which are a sub-Saharan African breed. It uses a MAG reconstruction strategy that several of the co-authors have previously used to great effect, which is having a very positive influence in the rumen microbiology/metagenomics field. Overall, this is a very well written and clearly presented paper, that to the best of my knowledge is technically sound. Given the quality of the methods used and written language, I have very little editorial comments to make here. However, that said, such MAG-centric approaches are very much (quickly) becoming standard practice in meta-omic studies. Therefore, I question the novelty of the information gained in this particular study that is limited in the number of animals used (6) and does not include any accompanying metadata or functional-omic data to assist in interpreting the metabolic contributions of the microbial populations sampled herein.

Some of the more interesting results include the higher proportion of taxa (such as the Lentisphaerae), although no annotative attempts (beyond CAZymes) are made to dig deeper into these genomes, or what their predicted functions and metabolic contributions to the rumen microbiome may be. It is also in this space that this study could have greatly benefited from some metadata (on fibers or sugars) or functional omic data (RNA or peptides) to help validate statements such as the one made on line 214: "This could indicate that their role in the rumen during periods of starvation may be more important when it is required for feed to undergo a greater degree of digestion to maximise energy conversion" or on lines 279-285 (e.g ….."linked to the use of molasses supplementation…")

I also question the value of MAG data being generated from 6 animals as a "vital resource" for future studies given we know the large influence variables such as diet, animal age or even individual genetics can have on microbiome structure. This variation means it is very likely that future studies would need to generate their own MAGs to ensure that their genomic diversity is sampled.

Minor comments

*	Line 81: The beginning of the results could benefit with some text into the samples that were used for sequencing, i.e. an expansion of the brief explanation of the experimental design that is made on Line 75

*	Line 163 «Comparison of MAG proteins to the dbCAN [19] CAZyme [20] database» This is not entirely accurate in its current form and implies that the CAZy database was used to generate these annotations. Rather, HMMs that are designed and made available by dbCAN are built using sequences that have been previously classified and catalogued by the CAZy team.

**Response to reviewers**

Reviewer 1:

This manuscript reported 1200 metagenome assembled genomes (MAGs) from the rumen of Zebu cattle under different dietary conditions. Although the identified MAGs do add more information to the current data pool of rumen microbiome, the current version is not suitable for being published at Genome Biology. The main issues are:

Q1. The concept is not new and novel. The work flow and contents are pretty similar to Stewart et al.(2018) except using rumen samples generated from different cattle.

We clearly stated and referred to the pipeline previously published and the novelty here is in the discovery of 1200 MAGs from the rumen of African livestock and their functional potentials in the context of feed restriction in harsh tropical environments. The new and novel metagenome-assembled genomes constitute a fundamental resource in particular as an unprecedented effort to 1) increase the currently very limited representation of the rumen microbiome from low- and middle-income countries in particular in Africa and to 2) investigate the changes in microbial abundance profiles in the context of sub-optimal feeding. The most comprehensive rumen microbiome databases reported to-date (Global Rumen Census [1], Hungate collection [2] , Rumen Uncultured Genomes [3,4]) still lack a fully complete geographical representation of the rumen worldwide with African countries being heavily under-studied. Additionally, very few studies report analysis at the level of metagenome-assembled genomes (MAGs), a field which is still at its infancy, none of which investigating the rumen microbial abundance profiles in relation to feed restriction. The discovery of new MAGs from animals living in those under-studied geographical regions is an unprecedented resource enabling a better understanding of the microbial populations that are thriving in harsh tropical environments. The findings reported here are new and novel and dramatically increase our knowledge of the microbial communities present within the rumen microbiome of African livestock alongside its functional potential.

We added some results and discussions to the manuscript to expand on the functional potential of the novel MAGs (L165-176), classify the CAZymes they encode and report their novelty (L193-205), and identify the contribution of the MAGs and their KEGG orthologs to key metabolic pathways of relevance to feed degradation and nutrition (L264-302). We identify the key drivers, both at MAG-level and KEGG orthologs-level, that contribute to the differential MAG abundance observed between diet treatments, and their associated functional potentials (L378-477).

Q2. Lack of fundamental biological questions and biological relevant interpretation of the results.

Q2a. The differential abundant ones had very low relative abundance. As described above, the manuscript does not have any new unanswered questions in terms of rumen microbiome.

We appreciate the reviewer’s comment and we have extended the manuscript to highlight the fundamental biological questions addressed emphasising the variation in microbial abundance between the diet treatments, showing clear distinctions associated with the lowest MER diet treatment (40%). We also extended the results section to include taxonomic analysis at a higher resolution (i.e. MAG-level) in addition to the original Phylum-level, and further expanded the discussion with the taxonomic classification of the significantly differentially abundant MAGs (Suppl. table S5 and L236-244). We report 87 and 47 significantly abundant MAGs between 40% vs. 80% and 60% vs. 80%, and their relative abundance ranging from 2e-4 to 3.6 with 36 (41%) and 26 (55%) MAGs showing relative abundance above 0.05 in the 40% and the 60% diets respectively. Additionally, the significantly differentially abundant MAGs have all been classified as members of taxonomic groups shown to be present and of relevance to the rumen environment (e.g. Prevotella, Clostridium, Ruminococcus).

We also highlight the new results along with new biological insights in 3 ways: 1) 1200 new MAGs identified and 850 dereplicated (at 99% ANI) with the majority (89%; 753/850) at most 90% similar to any other rumen genomes, 2) the functional annotations of those MAGs indicated by their CAZymes and KEGG-associated pathways highlights different MAG contributions underlying the distinct MER treatments and 3) this new MAG resource was further used to investigate MAG abundance profiles which highlighted novel and unprecedented insight into the differences in microbial abundance observed under sub-optimal feeding, and their contributions to key metabolic pathways within the rumen of Zebu cattle living in harsh tropical environments.

Q2b. It seems strange that the identified MAGs could not be found in known databases. It is very doubtful that the rumen microbiome of zebu cattle is completely different from those in other ruminants. Based on tremendous published 16S data and metagenome dataset, the difference between rumen microbiota of Bos Taurus and Bos Indicus do share many taxa. It is important to verify whether the identified novel MAGs are not just artifacts of computational based analysis.

We appreciate the reviewer’s comment and indeed our results confirm that the rumen microbiome of Zebu cattle is not completely different from those in other ruminants when looking at the community structure. Indeed, our work indicates that the rumen microbiome of Zebu cattle is similar in its taxonomical composition to that of the known rumen microbiome composition in other ruminants (e.g. high Pearson correlation coefficients: 0.99 and 0.98 versus RUG and RUG2 datasets respectively; see also table 2 and added text at lines L140-146).

Additionally, recent efforts, using cultured isolates, have highlighted the high level of genetic diversity within taxonomic groups of rumen organisms [5]. What our work also shows is the diversity of the genetic content observed at species level (i.e. at a high taxonomic resolution) with new and novel MAGs identified with a robust assemblybased discovery pipeline including stringent filtering parameters relating to genome completeness (>=80%) and contamination (<10%), based on single-copy marker genes as implemented in the checkM software [6]. The resulting high-quality genomes were used to investigate the effect of diet treatments.

Q2c. The identified significant differential abundant MAGs in response to diet are all extremely low abundant ones (less than 0.05%, Table 3). It is quite common for those low abundant ones to be significant different. Are these MAGs biological meaningful? Also, why the comparison is based on phylum level not lower taxonomic level?

As stated above in point Q2a, we also extended the results section to include taxonomic analysis at a higher resolution (i.e. MAG-level) in addition to the original Phylum-level, and further expanded the discussion with the taxonomic classification of the significantly differentially abundant MAGs (Suppl. Table S5 and L236-244). We report 87 and 47 significantly abundant MAGs between 40% vs. 80% and 60% vs. 80%, and their relative abundance ranging from 2e-4 to 3.6 with 36 (41%) and 26 (55%) MAGs showing relative abundance above 0.05 in the 40% and the 60% diets respectively.

Additionally, the significantly differentially abundant MAGs have all been classified as members of taxonomic groups shown to be present and of relevance to the rumen environment (e.g. Prevotella, Clostridium, Ruminococcus).

Q3. The sample size is small with only 6 cattle comparing to 43 in Stewart et al. (2018). The difference in abundances of MAGs under three dietary conditions were also just based on 6 cattle, which has very little power. Also, not clear why only three dietary conditions were compared, not four?

The section 6.1 referring to the sample collection and sequencing has been extended with additional description regarding the experimental design and the sequencing data we generated (L509-540). We collected and sequenced a total of 24 samples originating from 6 animals each fed 4 diets treatments based on a Latin square design. Sequencing on an Illumina HiSeq 4000 generated 749 Gb of data from 24 samples from the rumen of African Zebu cattle. The deep sequencing performed in our study resulted in a comparable amount of data to that reported in [3] (768 Gb of Illumina sequencing data). The differences in microbial abundances between diets were compared with animals being grouped per diet treatment. The 100% MER treatment was excluded from the comparative analysis of the effect of diet (section 3.5) as it contained a small amount of cottonseed and molasses which was not present in the other diet treatments -i.e. 40%, 60% and 80% MER- (see method section 6.1).

Additionally, several metagenomics studies investigating the functionality of the rumen in cattle and goat were based on few animals (up to 4 animals) per treatment/conditions [7–9] and have all helped in shaping our understanding of the rumen functionality.

Q4. Lack of descriptive information of sample sources which makes the results less convincing.

Based on Stewart et al.(2018), they generated 850 MAGs from 768Gb data. It is unclear how much sequence data were generated, if it is only 6 cattle with 106 million reads, the total amount of data was only 106MX150X6/1024M=93.16Gb. And how can such low about of data could generate more MAGs than Stewart et al.(2018)?

For clarity, as described in the above point 3, the section 6.1 referring to the sample collection and sequencing has been extended with additional description regarding the experimental design and the sequencing data we generated (L509-540). We collected and sequenced a total of 24 samples originating from 6 animals each fed 4 diets treatments based on a Latin square design. Sequencing on an Illumina HiSeq 4000 generated an average of 106 million 150bp paired-end reads per sample (or 212 million reads per sample) with a total of 749 Gb of data from 24 samples from the rumen of African Zebu cattle. The deep sequencing performed in our study resulted in a comparable amount of data to that reported in [3] (768 Gb of Illumina sequencing data), and we reconstructed a similar number of dereplicated winning MAGs, 850 vs 913 from Stewart et al 2018.

Q5. Lack of validation: All conclusions are based on bioinformatic analysis. For high impact journal like Genome Biology, the discovery in biological process is essential. There are some fundamental questions need to be answered through following up experiment. For example,

Q5a. Are all MAGs true colonizers? And do they only colonize in zebu? Are those novel MAGs having unique metabolic pathways and/or functions (such as detoxification) comparing to all known rumen cultures and MAGs? If so, will they be able to degrade different type of forages, for example, the forages only grown in Africa?

As stated above, the high level of correlation of taxonomic composition indicates that the MAGs we identify represent true colonisers of the rumen, representing some of both the core community and the variable community associated with variation in host and geographical range [1]. Genomes originating from the rumen of African zebu or other African ruminants are currently vastly under-represented in the public domain. While taxonomically they show correlation to other rumen organisms, until more genomes from a wider geographical and host range are sequenced it is impossible to say whether the sub-species present within our dataset are represented in other environments. Functional annotation resulted in a total of 3999 KOs identified with the 850 MAGs, 2675 of which are also found within the genomes from the Hungate collection. However, 1324 KOs from the MAG genomes were not found in Hungate genomes, 573 of which are related to metabolism. While these KOs do not represent novel pathways, they represent the variation in gene content seen within rumen organisms [5]. A further 260 KOs, not present in the Hungate genomes, were annotated as ‘poorly characterised’ within the KEGG database, while these are not novel, they serve to further highlight the lack of representation of certain environments within public databases. (L165-176)

Q5b. CAZymes: are there any of them novel enzymes? If so, can authors verify that they are actually produced in the rumen?

As stated above, we added some results to classify the CAZymes they encode and report their novelty (L193-219). We show that while all but one of the CAZyme families identified within the MAGs have been found within the RUG 2.0 genomes, the proteins in which the CAZyme domains are found are highly novel, with only 1286 out of 68,850 (1.8%) proteins showing complete identity to any protein within the nr, env_nr, M5nr, Uniprot TrEMBL databases or Hess et al, RUG or RUG 2.0 predicted protein datasets. Furthermore 53,838 of the 68,850 (78.2%) of the carbohydrate active proteins show less than 95% protein identity to any protein in these databases and datasets. Again, the robust assembly-based MAG discovery pipeline, would indicate that these proteins are truly encoded by the MAGs we identify.

Reviewer 2:

Q1. This paper presents 1200 high quality MAGs from 6 Zebu cattle, which are a sub- Saharan African breed. It uses a MAG reconstruction strategy that several of the coauthors have previously used to great effect, which is having a very positive influence in the rumen microbiology/metagenomics field. Overall, this is a very well written and clearly presented paper, that to the best of my knowledge is technically sound. Given the quality of the methods used and written language, I have very little editorial comments to make here. However, that said, such MAG-centric approaches are very much (quickly) becoming standard practice in meta-omic studies. Therefore, I question the novelty of the information gained in this particular study that is limited in the number of animals used (6) and does not include any accompanying metadata or functionalomic data to assist in interpreting the metabolic contributions of the microbial populations sampled herein.

We appreciate the reviewer’s comments and we have extended the manuscript with a whole new downstream analysis for the functional potential of the MAGs and their relevance in the context of the diet treatments (L165-176; L193-223; L236-244; L264- 302; L378-477).

Additionally, the section 6.1 referring to the sample collection and sequencing has been extended with additional description regarding the experimental design and the sequencing data we generated (L509-540). We collected and sequenced a total of 24 samples originating from 6 animals each fed 4 diets treatments based on a Latin square design. Sequencing on an Illumina HiSeq 4000 generated 749 Gb of data from 24 samples from the rumen of African Zebu cattle. The deep sequencing performed in our study resulted in a comparable amount of data to that reported in [3] (768 Gb of Illumina sequencing data). The differences in microbial abundances between diets were compared with animals being grouped per diet treatment. The 100% MER treatment was excluded from the comparative analysis of the effect of diet (section 3.5) as it contained a small amount of cottonseed and molasses which was not present in the other diet treatments -i.e. 40%, 60% and 80% MER- (see method section 6.1).

Additionally, several metagenomics studies investigating the functionality of the rumen in cattle and goat are based on few animals (up to 4 animals) per treatment/conditions [7–9] and have all contributed in shaping our understanding of the rumen functionality.

Q2. Some of the more interesting results include the higher proportion of taxa (such as the Lentisphaerae), although no annotative attempts (beyond CAZymes) are made to dig deeper into these genomes, or what their predicted functions and metabolic contributions to the rumen microbiome may be. It is also in this space that this study could have greatly benefited from some metadata (on fibers or sugars) or functional omic data (RNA or peptides) to help validate statements such as the one made on line 214: "This could indicate that their role in the rumen during periods of starvation may be more important when it is required for feed to undergo a greater degree of digestion to maximise energy conversion" or on lines 279-285 (e.g ….."linked to the use of molasses supplementation…")

We appreciate the reviewer’s comments and we added some downstream functional analysis in the results and discussions to the manuscript to expand on the functional potential of the novel MAGs (L165-176), classify the CAZymes they encode and report their novelty (L193-219), and identify the contribution of the MAGs and their KEGG orthologs to key metabolic pathways of relevance to feed degradation and nutrition (L264-302). We also identify the key drivers, both at MAG-level and KEGG orthologslevel, that contribute to the differential MAG abundance observed between diet treatments, and their associated functional potentials (L378-477).

Q3. I also question the value of MAG data being generated from 6 animals as a "vital resource" for future studies given we know the large influence variables such as diet, animal age or even individual genetics can have on microbiome structure. This variation means it is very likely that future studies would need to generate their own MAGs to ensure that their genomic diversity is sampled.

As stated above, we collected and sequenced a total of 24 samples originating from 6 animals each fed 4 diets treatments based on a Latin square design. Sequencing on an Illumina HiSeq 4000 generated 749 Gb of data from 24 samples from the rumen of African Zebu cattle. The deep sequencing performed in our study resulted in a comparable amount of data to that reported in [3] (768 Gb of Illumina sequencing data). Additionally, several metagenomics studies investigating the functionality of the rumen in cattle and goat are based on few animals (up to 4 animals) per treatment/conditions [7–9] and have all contributed in shaping our understanding of the rumen function.

The new and novel metagenome-assembled genomes (based on 24 rumen samples) constitute a fundamental resource in particular as an unprecedented effort to 1) increase the currently very limited representation of the rumen microbiome from lowand middle-income countries in particular in Africa and to 2) investigate the changes in microbial abundance profiles in the context of sub-optimal feeding. The most comprehensive rumen microbiome databases reported to-date (Global Rumen Census [1], Hungate collection [2] , Rumen Uncultured Genomes [3,4]) still lack a fully complete geographical representation of the rumen worldwide with African countries being heavily under-studied. Additionally, very few studies report analysis at the level of metagenome-assembled genomes (MAGs), a field which is still at its infancy, none of which investigating the rumen microbial abundance profiles in relation to feed restriction. The discovery of new MAGs from animals living in those under-studied geographical regions is an unprecedented resource enabling a better understanding of the microbial populations that are thriving in harsh tropical environments. The findings reported here are new and novel and dramatically increase our knowledge of the microbial communities present within the rumen microbiome of African livestock alongside its functional potential.

We present these 1200 MAGs as a metagenome resource that can be used to study the rumen of African cattle not as an exhaustive representation of the full genomic diversity of the rumen microbiome. We appreciate that there are known variations in microbial communities between individual animals, across diets and throughout ruminant development, what we provide here is a stepping stone into the rumen microbiome and its relevance in the context of sub-optimal feeding in African cattle.

Minor comments

*Line 81: The beginning of the results could benefit with some text into the samples that were used for sequencing, i.e. an expansion of the brief explanation of the experimental design that is made on Line 75

For clarity, the section 6.1 referring to the sample collection and sequencing has been extended with additional description regarding the experimental design and the sequencing data we generated (L509-540). We collected and sequenced a total of 24 samples originating from 6 animals each fed 4 diets treatments based on a Latin square design. Sequencing on an Illumina HiSeq 4000 generated an average of 106 million 150bp paired-end reads per sample (or 212 million reads per sample) with a total of 749 Gb of data from 24 samples from the rumen of African Zebu cattle. The deep sequencing performed in our study resulted in a comparable amount of data to that reported in [3] (768 Gb of Illumina sequencing data), and we reconstructed a similar number of dereplicated winning MAGs, 850 vs 913 from Stewart et al 2018

*Line 163 «Comparison of MAG proteins to the dbCAN [19] CAZyme [20] database» This is not entirely accurate in its current form and implies that the CAZy database was used to generate these annotations. Rather, HMMs that are designed and made available by dbCAN are built using sequences that have been previously classified and catalogued by the CAZy team.

Indeed, we addressed this comment and made appropriate changes at lines: L177- 178.

References

1. Henderson G, Cox F, Ganesh S, Jonker A, Young W, Janssen PH, et al. Rumen microbial community composition varies with diet and host, but a core microbiome is found across a wide geographical range. Sci Rep [Internet]. Nature Publishing Group; 2015 [cited 2019 Jul 8];5:14567. Available from:

http://www.nature.com/articles/srep14567

2. Seshadri R, Leahy SC, Attwood GT, Teh KH, Lambie SC, Cookson AL, et al. Cultivation and sequencing of rumen microbiome members from the Hungate1000 Collection. Nat Biotechnol. The Author(s); 2018;

3. Stewart RD, Auffret MD, Warr A, Wiser AH, Press MO, Langford KW, et al. Assembly of 913 microbial genomes from metagenomic sequencing of the cow rumen. Nat Commun [Internet]. Nature Publishing Group; 2018 [cited 2018 Mar 8];9:870.

Available from: http://www.nature.com/articles/s41467-018-03317-6

4. Stewart RD, Auffret MD, Warr A, Walker AW, Roehe R, Watson M. The genomic and proteomic landscape of the rumen microbiome revealed by comprehensive genome-resolved metagenomics. bioRxiv [Internet]. Cold Spring Harbor Laboratory; 2018 [cited 2019 Jun 25];489443. Available from:

https://www.biorxiv.org/content/10.1101/489443v1

5. Palevich N, Kelly WJ, Palevich N, Kelly W, Rakonjac J, Leahy S, et al. Pan-genome analysis of Butyrivibrio and Pseudobutyrivibrio provides insight into polysaccharide degradation in the rumen. 2015;

6. Parks DH, Imelfort M, Skennerton CT, Hugenholtz P, Tyson GW. CheckM: Assessing the quality of microbial genomes recovered from isolates, single cells, and metagenomes. Genome Res. 2015;

7. Wallace RJ, Rooke JA, McKain N, Duthie C-A, Hyslop JJ, Ross DW, et al. The rumen microbial metagenome associated with high methane production in cattle. BMC Genomics [Internet]. 2015 [cited 2017 Mar 7];16:839. Available from:

http://download.springer.com/static/pdf/7/art%253A10.1186%252Fs12864-015-2032-

0.pdf?originUrl=http%3A%2F%2Fbmcgenomics.biomedcentral.com%2Farticle%2F10.

1186%2Fs12864-015-2032-

0&token2=exp=1488905996~acl=%2Fstatic%2Fpdf%2F7%2Fart%25253A10.1186%2

5252Fs12864

8. Pitta DW, Pinchak WE, Indugu N, Vecchiarelli B, Sinha R, Fulford JD. Metagenomic Analysis of the Rumen Microbiome of Steers with Wheat-Induced Frothy Bloat. Front Microbiol [Internet]. Frontiers Media SA; 2016 [cited 2019 Jul 19];7:689. Available from:

http://www.ncbi.nlm.nih.gov/pubmed/27242715

9. Denman SE, Martinez Fernandez G, Shinkai T, Mitsumori M, McSweeney CS. Metagenomic analysis of the rumen microbial community following inhibition of methane formation by a halogenated methane analog. Front Microbiol [Internet]. Frontiers; 2015 [cited 2019 Jul 19];6:1087. Available from:

<http://journal.frontiersin.org/Article/10.3389/fmicb.2015.01087/abstract>

**2^nd^ round**

**Reviewer 1**

The revised version has improved significantly by including more analysis, especially the functional aspects. However, the main concern is still the small sample size as well as the experimental design to detect the dietary effect as well as the differential abundance analysis. It is still unclear how the findings from this study advanced the current knowledge in rumen microbiome. Although many new functions have been identified, it is unclear whether they belonged to novel MAGs, and if such functions have contributed to the rumen functional changes such as digestibility, VFAs, enzymatic activity and so on. The most importantly, if all these functional differences under different diets contribute to the phenotypic changes.

There was not power analysis performed to indicate that the number of samples has enough statistical power. Is it a 4X4 Latin Square design? If so, how the 6 animals were assigned for the 4 treatments. In addition, the Latin Square design is not proper for studying the rumen microbiome as it usually ignores the carry-over effect. There was no information about how long each treatment period was and how long was the adaptation period to minimize the carry over effect. In addition, it is unclear how Animal affects the MAGs. For Latin square design, it is important to collect rumen samples before the treatment to indicate the animal effect. Based on Table 7, the dietary effect could be confounded with animal effect if no baseline (before treatment) microbiome information was collected and analyzed.

Need to define the sub-optimal and optimal farming more clearly. What are the main issues to the animals under such farming conditions? Feed restriction based on energy requirement does not really represent harsh environments with food scarcity. The animals may eat very low-quality forage that can have low energy comparing to diet contains high protein, high starch even with restricted amount. The 850 MAGs were present in all samples, indicating that the diet has no effect on the richness at MAG level. This further confirms the limitation of this study in terms of nutritional treatments. The dietary effect on rumen microbiome should be minimized as the experimental design does not really reflect the dietary composition difference 3.5 and 3.5.1.

Comparison different abundance of MAGs and KOs (KO counts per MAG X abundance of MAG per each sample) under different diets. How the sequencing depth was taken into account? Would abundance of MAGs also depend on the sequencing depth? The estimation of the abundance of MAGs (Lines 218-219) does not seem to include such aspect. How was data normalized? This reflects the results outcomes as shown in section 3.5.1, although I do appreciate this section, but I have some concerns on the analysis. Based on M&M, the coverage estimates were corrected for contig length and GC content bias to produce a MAG abundance per sample matrix. This somehow does not take the sequence depth into consideration, so the differential abundance analysis can be largely biased.

Also, there is no phenotypic data. So, does the identified variation if abundance of MAGs and functions somehow contribute to the phenotypic differences? This is another important aspect that is missing from the revised version.

Other minor comments:

Introduction:

Lines 80-85: This newly added paragraph is not really relevant or specific enough to be relevant to this study.

Results:

Lines 132-133: suggest remove significantly, move the P value after each R value.

Line 138: please insert (Table 2) in the end of the sentence.

It is great to see the information of KOs and CAZymes included in the revised eversion. Can they be traced back to MAGs? The comparison of them with other reported databases are very useful, but it could be very helpful to see if the novel KOs and/or CAZymes belonged to the known MAGs from this and other studies or the novel MAGs identified from African Zebu cattle. Here, why the CAZyme distribution was analyzed at Phylum level? Also, are there any plans to validate some of the novel enzymes in the rumen?

Section 3.5: suggest including differential abundant MAGs as the result Table, while the phylum level (current Table 6) is better as supplementary. For both Verrucomicrobia and Synergistetes phyla, their abundances were extremely low (0.0004% to 0.04%)? For example, are these numbers meaningful? Most taxonomic study applies cut-off of 0.1% (some with 0.01%). In addition, did author consider the presence of any taxon in 4 out of 6 animals as detected taxa? So, the authors should justify whether these two phyla were truly detected in all six animals under one diet.

Discussions:

Again, lines 370-378, the advantages of the identifying at MAG level, is the better understanding of species/strain level and their contributions to the rumen microbiome functions. So, the discussions at phylum level is not meaningful.

Lines 399-404: kind of repeat M&M and results, not discussion.

Based on this report, we declined to publish the manuscript on the grounds of lack of clear advance, but offered the authors the opportunity to transfer the manuscript to an alternative journal. The authors appealed the decision.

**Author appeal**

We thank the editor, Dr Andrew Cosgrove, and the reviewer for their comments with regard to our original research article entitled “1200 High Quality Metagenome-Assembled Genomes from the Rumen of African Cattle and their relevance in the context of sub-optimal feeding”. We are writing the present letter to appeal against the decision to reject the manuscript from submission to Genome Biology as this decision was based on comments from a single reviewer that we have now strongly addressed (see below), and therefore we have also fully addressed all the concerns that the editor referred to when making this decision. We further demonstrated the relevance and novelty of our study and of its findings; and we have also included a new section with regard to power calculation clearly demonstrating the statistical power of this analysis, therefore confirming the relevance of the experimental design and of the results presented in this manuscript. Thus, having addressed all the points raised by the reviewer and the editor, we now call for re-consideration of the decision, and we would appreciate a rapid response within 14 days of reception of this mail with a straightforward acceptance of the final revised manuscript for submission in Genome Biology.

Please find below the point-by-point responses to the reviewer’s comments. We also provided the final revised manuscript (as attached document to this mail) with few amendments as described in our comments below, and an additional supplementary figure (Fig. S3) with detailed power calculation analysis.

**Reviewer 1**

The revised version has improved significantly by including more analysis, especially the functional aspects. However, the main concern is still the small sample size as well as the experimental design to detect the dietary effect as well as the differential abundance analysis. It is still unclear how the findings from this study advanced the current knowledge in rumen microbiome. Although many new functions have been identified, it is unclear whether they belonged to novel MAGs, and if such functions have contributed to the rumen functional changes such as digestibility, VFAs, enzymatic activity and so on. The most importantly, if all these functional differences under different diets contribute to the phenotypic changes.

We acknowledge that small sample sizes often do not provide enough statistical power to elucidate the effect associated with the conditions tested. However, as indicated by Debelius et al 2016, [1] precalculating power in microbiome studies is inherently problematic. This is because the taxon/OTU/MAG richness is needed to estimate effect size and therefore statistical power. Similarly, Nakagawa and Cuthill [2] highlight that power calculations place far too much emphasis on achieving statistical significance and that effect size is a far more useful metric to assess your experimental design. Power calculation analyses use the relationship between, power, sample size, effect size and significance value. The Adonis2 (PERMANOVA) analyses we include in section 3.5 used the diet as the grouping factor to test significance. When applying this to our MAG relative abundance data, an effect size of 44.6% (R2 = 0.446) is attributable to this diet. Similarly, when analysing the KO data in the same manner, an effect size of 45.9% (R2 = 0.459) is seen. Using the effect size calculated during the Adonis2 analyses, we calculated the statistical power associated with our experimental design. To assess the impact of different sample sizes on the statistical power of our data analysis, we used the R package micropower [3] to assess the impact of 3, 4 and 5 samples per treatment group (T40, T60 and T80 groups) as well as a 4:5:5 (T40:T60:T80) design to include the design used in this study. We used a random sampling with replacement approach to generate 1000 Euclidean distance matrices based on our data, for each of the specified sample size groupings (3:3:3, 4:4:4, 4:5:5 and 5:5:5 samples). Using the bootstrapped PERMANOVA power function within micropower we estimated the statistical power for each matrix based on 10,000 bootstrap permutations, for a total of 10 million estimates for each sample size grouping. This analysis was conducted for P values of 0.05, 0.01 and 0.001 and indicated that even at 0.001, the median power across 10 million permutations was 0.99 for the 4:5:5 design and 0.99 for the 5:5:5, but only 0.84 for the 4:4:4 design and no statistical power was associated with the 3:3:3 design (0.03). Analyses at 0.05 and 0.01 indicated powers of 1 for the 4:5:5 and 5:5:5 designs at both significance values. Therefore, the experimental design used in this study is clearly suited to detect microbial differences associated with the large effect size attributable to the diet treatment.

A section on power calculation (lines 672-692) and an additional supplementary figure (Figure S3) have been added to the manuscript to emphasise the statistical power associated with this analysis and the statistical significance of the results reported.

For all population and community analyses (Adonis2, CAP, PCA) raw counts were Hellinger transformed and batch corrected for animal and period effects using the R package limma [4]. This transformation represents the square root of the relative abundance and accounts for variations in library size and for the sparse matrices associated with microbiome studies.

As indicated in the DESeq2 documentation [5], “The DESeq2 model internally corrects for library size, so transformed or normalized values such as counts scaled by library size should not be used as input.” As such we used the raw read count matrix (Table S5) as input data during our DESeq2 analyses.

More detail has been added to the manuscript to cover this point at lines 635-647 and 655-658.

As demonstrated in our re-submitted manuscript, all 850 dereplicated MAGs identified in our study represent novel MAGs at 99% ANI. From all proteins encoded by these novel MAGs, 68,850 have carbohydrate active domains, 53,838 (78.2%) of these CAZy proteins show less than 95% identity to any protein in any other rumen database or dataset, representing 53,838 novel rumen-relevant proteins from our 850 novel MAGs. Similarly, of the 3999 KOs identified in these MAGs, 33% (1,324) were not found in genomes from the Hungate Collection, with 573 related to metabolism. Therefore 33% of KOs encoded by the 850 novel MAGs have not previously been seen in a rumen context.

Finally, we aimed to identify whether changes in the functional potential of the rumen microbiome could be detected as a result of dietary restriction. Our results indicate that this is indeed the case, and have provided insight into some of the rumen functionality that is affected, and while analysis of phenotypic changes within the animals (other than change in live weight) was beyond the scope of this experiment, our results provide a basis for rationalising the type of phenotypic changes to be assessed in future studies.

There was not power analysis performed to indicate that the number of samples has enough statistical power. Is it a 4X4 Latin Square design? If so, how the 6 animals were assigned for the 4 treatments. In addition, the Latin Square design is not proper for studying the rumen microbiome as it usually ignores the carry-over effect. There was no information about how long each treatment period was and how long was the adaptation period to minimize the carry over effect. In addition, it is unclear how Animal affects the MAGs. For Latin square design, it is important to collect rumen samples before the treatment to indicate the animal effect. Based on Table 7, the dietary effect could be confounded with animal effect if no baseline (before treatment) microbiome information was collected and analysed.

We appreciate the reviewer’s concerns and have added a whole section on power calculation, that clearly shows the high statistical power associated with our experimental design. As already outlined (see our reply to point 1 above), with very large effect sizes of 44.6% (MAG) and 45.9% (KO) attributable to the diet treatments, and an associated statistical power of over 99% (0.9914) we can conclude with greater than 99% confidence that the diet treatments have a clear impact on rumen community structure at a highly significant P value of 0.001.

Furthermore, the manuscript has been extended to include additional information regarding treatment and feed-up period length and sampling time points. Briefly, treatment periods were 35 days, including 14 days of adaptation. Prior to each treatment period, feed-up periods of 14 days at 100 MER diet were included to minimise carry-over. This two week ‘wash-out’ (feed-up) and two week adaptation period design is common in rumen microbiome studies [6], as is the Latin square and even incomplete Latin square experimental design [7–10].

We extended the manuscript at lines 528-532, 538-540 and 672-692 and also included an additional supplementary figure (Figure S3).

Need to define the sub-optimal and optimal farming more clearly. What are the main issues to the animals under such farming conditions? Feed restriction based on energy requirement does not really represent harsh environments with food scarcity. The animals may eat very low-quality forage that can have low energy comparing to diet contains high protein, high starch even with restricted amount. The 850 MAGs were present in all samples, indicating that the diet has no effect on the richness at MAG level. This further confirms the limitation of this study in terms of nutritional treatments. The dietary effect on rumen microbiome should be minimized as the experimental design does not really reflect the dietary composition difference 3.5 and 3.5.1.

Feed restriction is not a practice undertaken by smallholder farmers, but being able to feed their animals enough is a major concern. Here cattle in our experiment underwent feed restriction to simulate times when farmers have no access to animal feed. Basing this feed restriction on a ration calculated based on the animals’ live weight and therefore maintenance energy requirements (MER) is an experimental way of standardising the effect of restriction to allow thorough investigation into the effects of food scarcity on the rumen [11,12]. The experimental diet, made up of Rhodes grass hay, is already considered a low-quality forage and here the diet treatments we are investigating represent different extremes of food scarcity, represented by proportions of MER, and not variation in dietary composition.

Comparison different abundance of MAGs and KOs (KO counts per MAG X abundance of MAG per each sample) under different diets. How the sequencing depth was taken into account? Would abundance of MAGs also depend on the sequencing depth? The estimation of the abundance of MAGs (Lines 218-219) does not seem to include such aspect. How was data normalized? This reflects the results outcomes as shown in section 3.5.1, although I do appreciate this section, but I have some concerns on the analysis. Based on M&M, the coverage estimates were corrected for contig length and GC content bias to produce a MAG abundance per sample matrix. This somehow does not take the sequence depth into consideration, so the differential abundance analysis can be largely biased.

As previously highlighted (see our reply to point 1 above), for all population and community analyses (Adonis2, CAP, PCA) raw counts were Hellinger transformed and batch corrected for animal and period effects using the R package limma [4] This transformation represents the square root of the relative abundance and it accounts for variations in library size (sequencing depth) and for the sparse matrices associated with microbiome studies.

As indicated in the DESeq2 documentation: “The DESeq2 model internally corrects for library size, so transformed or normalized values such as counts scaled by library size should not be used as input.” As such we used the raw read count matrix (Table S5) as input data during our DESeq2 analyses.

More detail has been added to the manuscript to cover this at lines 635-647 and 655-658.

Also, there is no phenotypic data. So, does the identified variation if abundance of MAGs and functions somehow contribute to the phenotypic differences? This is another important aspect that is missing from the revised version.

Our study pioneers the investigation of the impact of sub-optimal feeding on the rumen microbiome in African cattle. Analysis of phenotypic changes within the animals (other than change in live weight) was beyond the scope of this experiment; here we aimed to identify whether changes in the functional potential of the rumen microbiome could be detected as a result of dietary restriction.

Introduction:

Lines 80-85: This newly added paragraph is not really relevant or specific enough to be relevant to this study.

We appreciate the reviewer’s comment. We removed this paragraph and added a summary sentence at lines 92-93.

Results: Lines 132-133: suggest remove significantly, move the P value after each R value.

We appreciate the reviewer’s comment, and amended the text accordingly at lines 139-140.

Line 138: please insert (Table 2) in the end of the sentence

We appreciate the reviewer’s comment, and amended the text accordingly at line 146.

It is great to see the information of KOs and CAZymes included in the revised eversion. Can they be traced back to MAGs? The comparison of them with other reported databases are very useful, but it could be very helpful to see if the novel KOs and/or CAZymes belonged to the known MAGs from this and other studies or the novel MAGs identified from African Zebu cattle. Here, why the CAZyme distribution was analyzed at Phylum level? Also, are there any plans to validate some of the novel enzymes in the rumen?

As demonstrated in our last revised manuscript, all 850 dereplicated MAGs identified in our study represent novel MAGs at 99% ANI. From all proteins encoded by these novel MAGs, 68,850 have carbohydrate active domains, 53,838 (78.2%) of these CAZy proteins show less than 95% identity to any protein in any other rumen database or dataset, representing 53,838 novel rumen-relevant proteins from our 850 novel MAGs. Similarly, of the 3999 KOs identified in these MAGs, 33% (1,324) were not found in genomes from the HungateCollection, with 573 related to metabolism. Therefore 33% of KOs encoded by the 850 novel MAGs have not previously been seen in a rumen context.

Table S4 and figure 7 contains the full distribution of CAZymes from all 850 novel MAGs, whereas Table S3 covers the full distribution of KOs across all 850 novel MAGs.

Section 3.5: suggest including differential abundant MAGs as the result Table, while the phylum level (current Table 6) is better as supplementary. For both Verrucomicrobia and Synergistetes phyla, their abundances were extremely low (0.0004% to 0.04%)? For example, are these numbers meaningful? Most taxonomic study applies cut-off of 0.1% (some with 0.01%). In addition, did author consider the presence of any taxon in 4 out of 6 animals as detected taxa? So, the authors should justify whether these two phyla were truly detected in all six animals under one diet.

The data presented in our study represents a metagenomic investigation into the rumen microbiome at extremely high sequencing depth (24 samples sequenced on HiSeq 4000 using 8 lanes). As indicated by Anantharaman et al. [13], deep sequencing at this scale reconstructs highly complete strain resolved genomes and allows tracking of dynamics in community composition and metabolic potential across an environment and also from the ‘rare biosphere’ which may represent less than 0.001% of a community. Here, the stringent thresholds applied for genome completeness and contamination (Fig 2) indicate that none of the identified MAGs could be considered spurious or an artefact of the data. Our mapping-based approach for abundance estimation resulted in a mean read count per MAG of 63,951 in the GC content and contig length corrected read count matrix (Table S5). The library size (total number of reads per sample) ranges from 35 million to 75 million reads and the distribution of maximum read count reported for a MAG in any sample ranges from 4,864 supporting reads to 6.7 million reads with a median of 238,820 reads; which clearly shows a very high support for the presence of these MAGs in the data and further supports the differential abundance analysis. Additionally, all 850 MAGs were found in all animals and all diets (statement clarified in manuscript at L228).

The high sequencing depth, stringent MAG quality criteria and high mapping rates provide evidence that all relative abundance comparisons are supported by large amounts of high-quality sequencing data, resulting in highly significant, biologically meaningful, comparisons of differential abundance across the diet treatments. Even if we apply a threshold of 0.001% abundance for MAG inclusion (the upper limit of the rare biosphere), our data still show significant differential abundance in the same 87 and 47 MAGs (T40 vs. T80 and T60 vs. T80 respectively), and also between the Synergistetes and Verrucomicrobia at Phylum level analysis, when comparing 40% diet to 80% diet.

Discussions: Again, lines 370-378, the advantages of the identifying at MAG level, is the better understanding of species/strain level and their contributions to the rumen microbiome functions. So, the discussions at phylum level is not meaningful.

The broad phylum-level rumen community structure comparison to 16S marker gene studies, highlights that our MAG abundance estimations are in line with what is already known about the rumen community structure. We agree that the higher resolution afforded by analysis at the MAG/taxon/OTU level better represents the contribution of taxonomic groups to the rumen function, and indeed we had included a detailed analysis at the MAG level as discussed in lines 391-483. The section covered by lines 382-390 further indicates that even at the much lower resolution of phylum level analyses, differences attributable to the diet treatments were evident.

Lines 399-404: kind of repeat M&M and results, not discussion.

This paragraph introduces the distinct scenarios observed and discussed in the next paragraphs and is therefore of relevance to the discussion.

References

1. Debelius J, Song SJ, Vazquez-Baeza Y, Xu ZZ, Gonzalez A, Knight R. Tiny microbes, enormous impacts: What matters in gut microbiome studies? Genome Biol. 2016.

2. Nakagawa S, Cuthill IC. Effect size, confidence interval and statistical significance: A practical guide for biologists. Biol. Rev. 2007.

3. Kelly BJ, Gross R, Bittinger K, Sherrill-Mix S, Lewis JD, Collman RG, et al. Power and sample-size estimation for microbiome studies using pairwise distances and PERMANOVA. Bioinformatics. 2015;31:2461–8.

4. Smyth GK. limma: Linear Models for Microarray Data. Bioinforma Comput Biol Solut Using R Bioconductor. 2005. p. 397–420.

5. Love M, Anders S, Huber W. Analyzing RNA-seq data with DESeq2. Bioconductor [Internet]. 2019; Available from: http://bioconductor.org/packages/devel/bioc/vignettes/DESeq2/inst/doc/DESeq2.html

6. Clemmons BA, Martino C, Schneider LG, Lefler J, Embree MM, Myer PR. Temporal Stability of the Ruminal Bacterial Communities in Beef Steers. Sci Rep. 2019;9:9522.

7. Kim SH, Mamuad LL, Kim EJ, Sung HG, Bae GS, Cho KK, et al. Effect of different concentrate diet levels on rumen fluid inoculum used for determination of in vitro rumen fermentation, methane concentration, and methanogen abundance and diversity. Ital J Anim Sci. 2018;17:359–67.

8. Petri RM, Kleefisch MT, Metzler-Zebeli BU, Zebeli Q, Klevenhusen F. Changes in the rumen epithelial microbiota of cattle and host gene expression in response to alterations in dietary carbohydrate composition. Appl Environ Microbiol. 2018;84.

9. McCann JC, Luan S, Cardoso FC, Derakhshani H, Khafipour E, Loor JJ. Induction of subacute ruminal acidosis affects the ruminal microbiome and epithelium. Front Microbiol. 2016;7.

10. Li R, Teng Z, Lang C, Zhou H, Zhong W, Ban Z, et al. Effect of different forage-to-concentrate ratios on ruminal bacterial structure and real-time methane production in sheep. PLoS One. 2019;14.

11. Grimaud P, Richard D, Vergeron MP, Guilleret JR, Doreau M. Effect of Drastic Undernutrition on Digestion in Zebu Cattle Receiving a Diet Based on Rice Straw. J Dairy Sci. 1999;82:974–81.

12. Grimaud P, Doreau M. Effects of level of intake and nitrogen supplementation on digestion by cows in a tropical environment. Anim Res. 2003;52:103–18.

13. Anantharaman K, Brown CT, Hug LA, Sharon I, Castelle CJ, Probst AJ, et al. Thousands of microbial genomes shed light on interconnected biogeochemical processes in an aquifer system. Nat Commun. 2016;7.

**Reviewer 1:**

After some research, I don't think the authors provided proper justification on what they did. Again, I do still have concern on their Latin square which was incorrect (please see below), and which actually affected their power of the metagenomic data significantly. Secondly, their power analysis does not tell that they have used appropriate numbers. It is really hard to achieve an effect size of 0.4 through metagenome data, which required 2/3 of the animals in the control group to be lower/higher than the average of the treatment group.

Specifically:

Assigning 6 animals to a 4 X 4 Latin square design is statistically not acceptable. If the authors check the design clearly, 2 of diet had 2 replicates in all 4 periods while 2 of the diet only had 1 animal in one period. I consulted this with well known dairy nutritionists, both of them agree that the experimental design was not balanced at all to reveal the treatment effect. I think authors first need to balance the replications for each diet under each time period. In reality it needs to have n=4 for the statistical analysis for each time point

It seems authors used micropower package reported by Kelly et al., 2015 to perform a power analysis. This power analysis generates effect size as ω2-value that ranged from 0-1. It is important that authors understand the use of effect size correctly. A 0 effect size represents that 50% of animals in the control need to be below the mean/average of the treatment group. Authors present an effect size of 0.4 (I am skeptical of this value as it is presented as R2). An effect size of 0.4 represents that 66% of control is below the average of the treatment. Is it possible to achieve such an effect size by using 4 animals? In Kelly et al, it reports that an n=5 will have 90% power to detect a ω2 of 0.05. So, here it is clear the effect size is fairly low (close to 0). Thus, I strongly believe that it is equally important that authors perform appropriate analysis as well as accurately interpret the results. Besides the power calculation information is not clearly presented under the methods which is misleading to showcase the true power.

However, as I am not an expert in statistics, I may be biased, although I think I am correct based on the above research. To be fair to the authors, please consult with the experts in the field which may give you more insights on their statistical power.

Regardless, I do agree the bioinformatic work from the same group is excellent, and many of us are following. However, the lack of understanding of Animal Biology may be a continuing concern on their experimental design to draw the biologically relevant conclusions. Maybe their advanced bioinformatic approach is still considerable for the Journal, but then they need to revise their conclusions.

We sought two new reviewers to comment on the statistical analysis.

**Reviewer 3**

I've had some time to look at the paper and the correspondence. Please, keep in mind that I am not an expert on the subject (cattle microbiome and diet) and hence I cannot comment on the appropriateness of the length of the "wash-out" period on the carry-over effect. That said, I think that there are two separate issues here, namely (1) the appropriateness of the design and (2) the power of the study to detect differences. I'll try to summarize my thoughts on both below, but in general I think that (1) is much more important than (2).

(1) Design. The reviewer raises concerns on the appropriateness of the design. The authors claim to use a Latin Square design, but by definition a Latin Square must be a "square" (i.e., based on a n by n table -- or multiples of n), while here they have n=6 animals and only 4 periods. This, as noted by the reviewer, leads to an unbalanced design with respect to period (while it is balanced with respect to the animals as each animal experiences all diets). This should be recognized by the authors and the design should be referenced as crossover but not as a Latin Square. The imbalance of the design is increased by the choice of the authors to remove all samples from the first period and all the samples with 100% diet. In particular, removing the first period leads to an imbalance with respect to animal as now not all animals will experience all diets. Again, I'm not an expert on cattle feeding, but my feeling is that the authors did not justify enough the sample exclusions. This type of post-hoc exclusions are often problematic. E.g., if the reason to exclude the samples in the first period is the difference in sample preparation, the inclusion of a "sample preparation" covariate in the model might be better than the exclusion of the samples. While balanced designs are preferable, this does not mean that unbalanced designs cannot lead to valid conclusions. DESeq2 can certainly handle this type of design, but the authors need to give more details on the model specification (e.g., the design matrix used) and the test that they performed. For instance, was an animal effect included in the model? Sharing the actual R code would be even better.

(2) Power. I agree with the authors that it is difficult to perform power analyses in high-throughput / high dimensional studies. Frankly, I am not sure about the importance of power in this type of experiments. Since the emphasis is often in what is found significant, even if the study is underpowered it could still be possible to identify those differential abundant features that have a high enough effect size. I am not familiar with the micropower package and I would need more time to look at the details of the approach to understand what the authors and reviewer refer to, but my main concern is whether, in the power analysis that the authors performed, the subjects were considered independent. In their design they clearly are not, as the same animal is fed all the diets, so an animal effect must be included in the model. This will affect power.

Additional point:

When the authors discuss the differential abundance results in the text, they talk about "highly significant" results (P<0.001). Were these p-values corrected for multiple testing? How? These details should be added in the text.

**Reviewer 4**

The reviewer seems to be concerned with two main issues: 1) design of the Latin square is unbalanced, hence time effect can confound treatment effect, and 2) sample size is too small.

I think 1) is a valid point. In Table 7, we observe that treatment 40 is assigned once for time period 2 and 3, and twice for 1 and 4, whereas treatment 60 is assigned once for period 1 and 2, and twice for 3 and 4. So intuitively, if there’s microbial difference between time periods 1 and 3 (where the design differed), then observed difference between treatments 40 and 60 can be attributed to this.

I don’t have enough knowledge on Latin square designs to advise canonical ways to address this. The reviewer’s suggestion to increase replicates to balance the design seems difficult to implement though. For statistical analyses, a multivariate, adjusted approach usually corrects for confounding, and I don’t see strong reasons why it won’t work here (to clarify, for a perfectly balanced Latin square multivariate models are unnecessary). For reference see e.g., https://www.ncbi.nlm.nih.gov/pmc/articles/PMC4017459/. Specifically, for differential abundance analysis, the authors can include time period as a covariate. For PERMANOVA, the authors can use distance ~ time period + treatment rather than distance ~ treatment (note the order of covariate is important in the new model, i.e. time period first treatment second). The suggested PERMANOVA analysis can err on the side of conservative, but hopefully can help with the reviewer’s concern.

For 2) I tend to side with the authors. I think the reviewer is concerned that the sample size is too small to detect small effect sizes, which is a valid statement in general. However the authors reported really large effect sizes here (>40%), which, checking the n=5 case in Kelly et al. Fig. 3, does correspond to high power (close to 1). I also don’t necessarily agree with the reviewer’s comment that “an effect size of 40% to represents that 66% of control is below the average of the treatment”. I’d interpret a PERMANOVA w^2 of 40% more towards “40% of the variability in samples’ overall microbial compositions can be attributable to treatment”.

That being said, is it suspicious that the effect size is so extreme (and correspondingly, the perfectly clustered samples in Figure 8)? From a biological point of view, does it make sense that diet has such a huge effect on samples’ microbiome? This I’ll defer to your and other reviewers' expertise. From a statistical point of view, I’m quite surprised that PERMANOVA and Figure 8 suggest such drastic difference between treatments, whereas the phylum (Table 6) or MAG (line 227-236) level differential abundance testing results are much more moderate (phylum composition seems similar, <10% MAGs significantly different). Admittedly this is more stemmed from my own concern, so please feel free to disregard and defer to the actual reviewers’s comments :). But I do think it worth investigating, independent of the sample size / power discussion.

**Author response**

We would like to thank both the editor and the two statisticians for their valuable comments with regard to our original research article entitled “1200 High Quality Metagenome-Assembled Genomes from the Rumen of African Cattle and their relevance in the context of sub-optimal feeding”.

Please find below the point-by-point responses to these comments. We also provided the final revised manuscript (as attached document to this mail) with few amendments as described in our comments below. We have also provided a link to download the additional R script as requested and the overall set of figures and tables, some of which with updated numbering as referred to in the final revised manuscript.

Editor Comment: Additional Reviewer 1 also comments on the exclusion of subjects from the analysis, and says that it would be preferable to include the reason for exclusion as a covariate; and also whether subjects were considered as independent in the analysis. This reviewer also asks about multiple testing correction – from my reading of your methods section, it appears that you did this, but it might be worth clarifying that in the main body of the text where appropriate.

As we discuss in the section at lines 512-525, we believe that due to the differences in sample storage, it is more appropriate for the samples to be removed from downstream analysis with an additional reference [43, Pollock et al 2018] which states: “Consequently, when considering the use of a cryoprotectant for storage, it is important to ensure that all samples are stored in the same manner.”

Similarly, as dietary composition is known to be the main driver of microbial differences in the rumen, it is more appropriate that these samples are removed form downstream analysis, especially as the dietary additive is confounded with the 100% MER diet (additional information added at lines 519-525).

It was not required for subjects to be considered as independent, as the batch correction we applied accounts for animal and period effects. More information was added at lines: 237-238, 243-244, 617 and 655-657.

Editor comment: Additional Reviewer 2 comments on the large effect on the microbiome caused by the change in diet, and that there is a relatively small change in phylum abundance. It seems to me that these results are not unexpected, but they may be worth commenting on in the text.

Indeed, these results are not unexpected, as analysis at phylum level represents an aggregation of counts belonging to all MAGs from a representative phylum, and therefore a lower resolution, it is not surprising that only small effects are seen at this level. The statistics are performed at MAG level and are therefore not representative of the changes seen here.

Editor comment: Since both reviewers have raised concerns about the way covariates were handled in the PERMANOVA, we ask you to address these concerns with one final revision. I appreciate that you do not want to perform further revisions, but it seems to us that these will be relatively straightforward to implement, and will strengthen the manuscript. It is possible that your analysis already takes some of these concerns into account in any case, but in which case this needs to be made clearer in the manuscript – both of the additional reviewers comment on the lack of detail about the PERMANOVA. Additional Reviewer 1 mentions that the code should be made available, and that would be a requirement of us accepting the manuscript.

Indeed, we appreciate the editor’s feedback and we have addressed these points and performed the final updates (as described in our responses to the reviewers’ comments below).

**Reviewer 3**

Design. The reviewer raises concerns on the appropriateness of the design. The authors claim to use a Latin Square design, but by definition a Latin Square must be a "square" (i.e., based on a n by n table -- or multiples of n), while here they have n=6 animals and only 4 periods. This, as noted by the reviewer, leads to an unbalanced design with respect to period (while it is balanced with respect to the animals as each animal experiences all diets). This should be recognized by the authors and the design should be referenced as crossover but not as a Latin Square.

Indeed, we appreciate the reviewer’s comment and we have edited the text to reflect this, removing reference to Latin square (Lines 491, 504 and 508).

The imbalance of the design is increased by the choice of the authors to remove all samples from the first period and all the samples with 100% diet. In particular, removing the first period leads to an imbalance with respect to animal as now not all animals will experience all diets. Again, I'm not an expert on cattle feeding, but my feeling is that the authors did not justify enough the sample exclusions. This type of post-hoc exclusions are often problematic. E.g., if the reason to exclude the samples in the first period is the difference in sample preparation, the inclusion of a "sample preparation" covariate in the model might be better than the exclusion of the samples. While balanced designs are preferable, this does not mean that unbalanced designs cannot lead to valid conclusions.

As mentioned above, in our responses to the editor’s comments: as we discuss in the section at lines 512- 525, we believe that due to the differences in sample storage, it is more appropriate for the samples to be removed from downstream analysis with an additional reference [43, Pollock et al 2018] which states: “Consequently, when considering the use of a cryoprotectant for storage, it is important to ensure that all samples are stored in the same manner”. Similarly, as dietary composition is known to be the main driver of microbial differences in the rumen, it is more appropriate that these samples are removed form downstream analysis, especially as the dietary additive is confounded with the 100% MER diet (additional information added at lines 519-525). It was not required for subjects to be considered as independent, as the batch correction we applied accounts for animal and period effects. More information was added at lines: 237-238, 243-244, 617 and 655-657.

DESeq2 can certainly handle this type of design, but the authors need to give more details on the model specification (e.g., the design matrix used) and the test that they performed. For instance, was an animal effect included in the model? Sharing the actual R code would be even better.

As discussed above, due to findings in previous studies (Pollock 2018 and Henderson 2015) we believe that it is more appropriate to remove samples representing a different experimental protocol and also those consisting of a different dietary composition that are confounded with our intended investigation into dietary amount (% MER) and the effect on rumen microbial abundance and function. Additionally, as the DESeq2 results are discussed extensively and compared across MAG and KO level data, we have revisited this analysis to use the more stringent likelihood ratio test (LRT) within DESeq2 (vs. the Wald test as used previously). Despite the more conservative nature of the LRT approach, we still see the same three scenarios as previously where MAG and KO level analyses highlight a more complex nature of the response of the rumen to dietary change. Updated methodology, results and discussion for this analysis are included at lines 221-228, 251-259, 269- 287, 372-376, 379-390, 394, 398-437, 627-632, 640-642, 679; and in relevant tables S9, S10 and S11 and their associated legends at lines 1001-1021. As requested, the R script containing the code to perform DESeq2 analyses has been included, as Additional file 1, and is referenced at lines 631-632 and 641-642 with associated legend at lines 1023-1029. Additionally, all input tables for DESeq2 and phyloseq analyses required as inputs in the R script provided (Additional File 1) have been included as Table S5 (updated format for analysis), and additional tables S6, S7 and S8 and their relevant legends at lines: 989-999.

Power. I agree with the authors that it is difficult to perform power analyses in high-throughput / high dimensional studies. Frankly, I am not sure about the importance of power in this type of experiments. Since the emphasis is often in what is found significant, even if the study is underpowered it could still be possible to identify those differential abundant features that have a high enough effect size. I am not familiar with the micropower package and I would need more time to look at the details of the approach to understand what the authors and reviewer refer to, but my main concern is whether, in the power analysis that the authors performed, the subjects were considered independent. In their design they clearly are not, as the same animal is fed all the diets, so an animal effect must be included in the model. This will affect power.

As previously mentioned, it was not required for subjects to be considered as independent as the batch correction we applied accounts for animal and period effects. More information was added at 237-238, 243-244, 617 and also at lines 655-657 relating specifically to the power analyses.

Additional point: When the authors discuss the differential abundance results in the text, they talk about "highly significant" results (P<0.001). Were these p-values corrected for multiple testing? How? These details should be added in the text.

DESeq2 corrects for multiple testing as standard using Benjamini-Hochberg (FDR), p-values given are already adjusted (please see text in the methods section at line 619).

**Reviewer 4**

The reviewer seems to be concerned with two main issues: 1) design of the Latin square is unbalanced, hence time effect can confound treatment effect, and 2) sample size is too small.

I think 1) is a valid point. In Table 7, we observe that treatment 40 is assigned once for time period 2 and 3, and twice for 1 and 4, whereas treatment 60 is assigned once for period 1 and 2, and twice for 3 and 4. So intuitively, if there’s microbial difference between time periods 1 and 3 (where the design differed), then observed difference between treatments 40 and 60 can be attributed to this.

As we discuss in the section at lines 512-525, we believe that due to the differences in sample storage, it is more appropriate for the samples to be removed from downstream analysis with an additional reference [43, Pollock et al 2018] which states: “Consequently, when considering the use of a cryoprotectant for storage, it is important to ensure that all samples are stored in the same manner.”

Similarly, as dietary composition is known to be the main driver of microbial differences in the rumen, it is more appropriate that these samples are removed form downstream analysis, especially as the dietary additive is confounded with the 100% MER diet (additional information added at lines 519-525).

It was not required for subjects to be considered as independent, as the batch correction we applied accounts for animal and period effects. More information was added at lines: 237-238, 243-244, 617 and 655-657.

I don’t have enough knowledge on Latin square designs to advise canonical ways to address this. The reviewer’s suggestion to increase replicates to balance the design seems difficult to implement though. For statistical analyses, a multivariate, adjusted approach usually corrects for confounding, and I don’t see strong reasons why it won’t work here (to clarify, for a perfectly balanced Latin square multivariate models are unnecessary). For reference see e.g., https://www.ncbi.nlm.nih.gov/pmc/articles/PMC4017459/. Specifically, for differential abundance analysis, the authors can include time period as a covariate. For PERMANOVA, the authors can use distance ~ time period + treatment rather than distance ~ treatment (note the order of covariate is important in the new model, i.e. time period first treatment second). The suggested PERMANOVA analysis can err on the side of conservative, but hopefully can help with the reviewer’s concern.

As mentioned above, we have revisited this analysis to use the more stringent likelihood ratio test (LRT) within DESeq2 (vs the Wald test as used previously). It was not required for subjects to be considered as independent, as the batch correction we applied accounts for animal and period effects. More information was added at lines: 237-238, 243-244, 617 and 655-657.

For 2) I tend to side with the authors. I think the reviewer is concerned that the sample size is too small to detect small effect sizes, which is a valid statement in general. However the authors reported really large effect sizes here (>40%), which, checking the n=5 case in Kelly et al. Fig. 3, does correspond to high power (close to 1). That being said, is it suspicious that the effect size is so extreme (and correspondingly, the perfectly clustered samples in Figure 8)?

To ease interpretation, we have now included the unconstrained PCoA plot as Figure 8 in the manuscript (in place of the Canonical Analysis of Principal coordinates i.e. CAP plot). The naïve nature of PCoA ordination contains all variance incorporated in the data, which similarly shows distinct clustering of samples belonging to the 40%, 60% and 80% diets, emphasising the large effect size we see in PERMANOVA analysis relating to the Diet factor. The CAP plot, which was previously included in the manuscript, was constrained with Diet as the factor of interest highlighting variance and clustering associated with this factor. Updated methodology and results can be found at lines: 239-241, 376, 619-620; figure 8 and its associated legend at lines 935-937.

From a biological point of view, does it make sense that diet has such a huge effect on samples’ microbiome? This I’ll defer to your and other reviewers' expertise.

As discussed above, diet has been shown to be the main driver of microbial change (Henderson 2015), so as such it is not that surprising to observe huge effects on samples’ microbiomes. Our study pioneers the investigation of the impact of sub-optimal feeding on the rumen microbiome in African cattle. Here we assessed the microbial differences associated with distinct amounts of %MER rather than distinct dietary compositions, and the observations relate to changes in microbial abundances between diet treatments as opposed to presence/absence of specific communities. The results are very interesting in that variable amounts of a given diet have a large impact on rumen microbial abundances, in addition to the known effect of diet itself as a primary driver of microbial change.

From a statistical point of view, I’m quite surprised that PERMANOVA and Figure 8 suggest such drastic difference between treatments, whereas the phylum (Table 6) or MAG (line 227-236) level differential abundance testing results are much more moderate (phylum composition seems similar, <10% MAGs significantly different). Admittedly this is more stemmed from my own concern, so please feel free to disregard and defer to the actual reviewers’s comments :). But I do think it worth investigating, independent of the sample size / power discussion.

As analysis at phylum level represents an aggregation of counts belonging to all MAGs from a representative phylum, and therefore a lower resolution, it is not surprising that only small effects are seen at this level. The statistics are performed at MAG level and are therefore not representative of the changes seen here. Please note DESeq2 results only report significantly differentially abundant MAGs, there are many differences in abundance that are not significant on their own but which add up to an overall significant difference in the microbial community.
